# Mutations of the DNA repair gene *PNKP* in a patient with microcephaly, seizures, and developmental delay (MCSZ) presenting with a high-grade brain tumor

**DOI:** 10.1038/s41598-022-09097-w

**Published:** 2022-03-30

**Authors:** Bingcheng Jiang, Cameron Murray, Bonnie L. Cole, J. N. Mark Glover, Gordon K. Chan, Jean Deschenes, Rajam S. Mani, Sudip Subedi, John D. Nerva, Anthony C. Wang, Christina M. Lockwood, Heather C. Mefford, Sarah E. S. Leary, Jeffery G. Ojemann, Michael Weinfeld, Chibawanye I. Ene

**Affiliations:** 1 Department of Oncology, University of Alberta, Cross Cancer Institute, 11560 University Ave., Edmonton, AB T6G 1Z2 Canada; 2 Department of Biochemistry, University of Alberta, Medical Sciences Building, Edmonton, AB T6G 2H7 Canada; 3 Department of Pathology, University of Washington, Seattle, WA USA; 4 Department of Laboratory Medicine and Pathology, University of Alberta, Cross Cancer Institute, 11560 University Ave., Edmonton, AB T6G 1Z2 Canada; 5 Department of Neurological Surgery, Tulane University, New Orleans, LA USA; 6 Department of Neurological Surgery, University of California Los Angeles, Los Angeles, CA USA; 7 Department of Laboratory Medicine, University of Washington, Seattle, WA USA; 8 Division of Genetics Medicine, University of Washington, Seattle, WA USA; 9 Division of Pediatric Hematology/Oncology, Seattle Children’s Hospital, Seattle, WA USA; 10 Department of Neurological Surgery, University of Washington, Seattle, WA USA; 11 Department of Neurological Surgery, MD Anderson Cancer Center, Houston, TX USA

**Keywords:** Biochemistry, Cancer, Genetics

## Abstract

Polynucleotide Kinase-Phosphatase (PNKP) is a bifunctional enzyme that possesses both DNA 3′-phosphatase and DNA 5′-kinase activities, which are required for processing termini of single- and double-strand breaks generated by reactive oxygen species (ROS), ionizing radiation and topoisomerase I poisons. Even though PNKP is central to DNA repair, there have been no reports linking PNKP mutations in a Microcephaly, Seizures, and Developmental Delay (MSCZ) patient to cancer. Here, we characterized the biochemical significance of 2 germ-line point mutations in the PNKP gene of a 3-year old male with MSCZ who presented with a high-grade brain tumor (glioblastoma multiforme) within the cerebellum. Functional and biochemical studies demonstrated these PNKP mutations significantly diminished DNA kinase/phosphatase activities, altered its cellular distribution, caused defective repair of DNA single/double stranded breaks, and were associated with a higher propensity for oncogenic transformation. Our findings indicate that specific PNKP mutations may contribute to tumor initiation within susceptible cells in the CNS by limiting DNA damage repair and increasing rates of spontaneous mutations resulting in pediatric glioma associated driver mutations such as ATRX and TP53.

## Introduction

Mutations in *PNKP* have been found to be responsible for three different relatively rare neurological diseases: an autosomal recessive neurodegenerative disease Ataxia-ocular motor apraxia 4 (AOA4)^1–6^, a variant of the hereditary peripheral neuropathy Charcot–Marie–Tooth disease (CMT2B2)^7^ and the autosomal recessive neurodevelopmental disorder Microcephaly, seizures, and developmental delay (MCSZ)^5,8–12^.

Neuronal cells are sensitive to DNA damage, especially endogenous DNA damage, due to their long life-span. Recent studies have highlighted the important involvement of DNA strand break processing enzymes in maintaining the genetic stability of neuronal cells^13,14^. One enzyme that plays a critical role in DNA strand break processing is polynucleotide kinase/phosphatase (PNKP). Human PNKP is a 57.1-kDa bifunctional enzyme that possesses both DNA 3′-phosphatase and DNA 5′-kinase activities, which are required for processing strand break termini of single- and double-strand breaks generated by reactive oxygen species (ROS), ionizing radiation and topoisomerase I poisons DNA^15,16^. Mammalian PNKP protein consists of an N-terminal forkhead-associated (FHA) domain and a catalytic subunit, attached by a flexible linker^17^. The FHA domain interacts with the phosphorylated forms of the scaffold proteins, X-ray repair cross-complementing protein 1 (XRCC1)^18,19^ and X-ray repair cross-complementing protein 4 (XRCC4)^20^, key components of single-strand break repair (SSBR) and the non-homologous end joining pathway (NHEJ) for repair of double-strand breaks, respectively. These interactions, facilitated by the FHA domain, help direct PNKP to the sites of DNA damage^16,17^. The catalytic subunit includes the phosphatase domain and the C-terminal kinase domain, which are tightly associated with each other both in terms of structure and function^17^.

Mutations in *PNKP* have been found to be responsible for three different relatively rare neurological diseases: an autosomal recessive neurodegenerative disease Ataxia-ocular motor apraxia 4 (AOA4)^1–6^, a variant of the hereditary peripheral neuropathy Charcot–Marie–Tooth disease (CMT2B2)^7^ and the autosomal recessive neurodevelopmental disorder Microcephaly, seizures, and developmental delay (MCSZ)^5,8–12^. To date MCSZ has been diagnosed in fewer than 30 families worldwide. It has the following clinical features: microcephaly, infantile-onset seizures, developmental delay and behavioral problems^5,8–12^. Mutation sites of *PNKP* have been found in all three domains—FHA, phosphatase and kinase—in these cases of MCSZ^5,8–12,21^. One patient with AOA4 was diagnosed with cerebellar pilocytic astrocytoma at the age of 23 years^22^, and a second AOA4 patient developed a cerebellar hemangioblastoma resulting from a concurrent mutation in the *von Hippel-Lindau* gene^23^, but until now no cancer has been reported in an MCSZ patient.

Here, we report on a male MCSZ patient presenting with a cerebellar high-grade brain tumor, glioblastoma multiforme (GBM). The cerebellar GBM was discovered when he was 3 years old. Genetic screening for mutations associated with his clinical features showed that he carried 2 newly discovered somatic *PNKP* point mutations. The two *PNKP* mutations found in the patient are C302T, which causes a proline to leucine change at position 101 (P101L) in the FHA domain and C968T, which causes a threonine to methionine change at position 323 (T323M) in the phosphatase domain of PNKP protein. While the latter mutation has recently been identified in a Dutch MCSZ patient^23^, the other *PNKP* mutation has not been found in any reported MCSZ/AOA4/CMT2B2 patients before. Both mutations are found in highly conserved regions of *PNKP*. Furthermore, this is the first reported case of an MCSZ patient to develop cancer. The primary focus of our study was to determine the biochemical and cellular consequences of these *PNKP* mutations during DNA damage repair and oncogenic transformation.

## Results

### Clinical presentation and histopathological features of brain tumor

**Figure 1:**
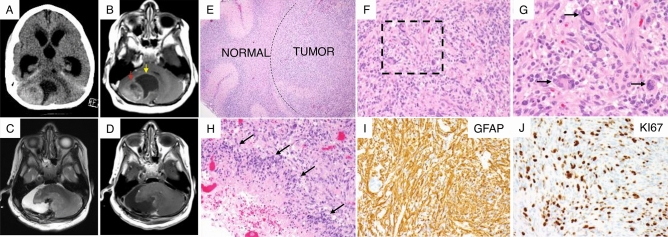
Clinical presentation and tumor histology. (**A**) CT scan of the head demonstrating large cerebellar tumor causing obstructive hydrocephalus. (**B**) T1-weighted MRI scan of the brain with contrast showing a heterogenous lesion (red arrow) alongside large cyst (yellow arrow) within the cerebellum. (**C**,**D**) T2-weighted (**C**) and T1-contrast enhanced MRI scan (**D**) of the brain post-surgery showing near total resection of the tumor. (**E**) Lower magnification hematoxylin and eosin (H&E) stain of tumor tissue showing expansion and infiltration of normal cerebellum (left) by tumor cells (right). (**F**) Higher magnification H&E stain of tumor demonstrating nuclear pleomorphism (aggressive tumor feature). (**G**) Higher magnification H&E stain (of dashed box in **F**) showing nuclear pleomorphism (black arrows). (**H**) The tumor also demonstrates pseudopalisading necrosis (black arrows), a histopathological feature of glioblastoma multiforme. (**I**,**J**) Immunohistochemistry showing glial origin for tumor based on glial fibrillary acid protein (GFAP) expression (**I**) and a highly proliferative lesion based on approximately > 30–40% Ki-67 antigen expression in tumor cells imaged at medium power (**J**).

The patient was a 3-year-old male with a history of known *PNKP* mutations resulting in medically intractable epilepsy, global developmental delay and microcephaly. Developmentally, at age 2, he had not yet crawled or pulled to a stand, although he was able to sit independently and roll to get on his hands and knees. At 3 years, he had not yet spoken any words and did not understand any words spoken to him. Now, he presented with worsening seizures, difficulty feeding and failure to thrive. A head computed tomography (CT) scan demonstrated a 2.5-cm hyper-dense lesion within the right cerebellum with evidence of obstructive hydrocephalus (Fig. 1A). An MRI of the brain demonstrated a 3 × 2.4 cm contrast enhancing lesion within the right cerebellum with a large 3.3 × 2.7 cm peri-tumoral cyst (Fig. 1B). The patient was taken to the operating room for a near-total resection (NTR) of the cerebellar mass without complications (Fig. 1C,D).

Histological sections of the tumor tissue demonstrated a highly cellular neoplastic proliferation of intermediate to large sized pleomorphic cells (Fig. 1E–G). There were also areas of pseudopalisading necrosis indicating the highest grade of glioma (Fig. 1H). Immunohistochemistry demonstrated GFAP positivity suggesting glial tumor (Fig. 1I*)* and a Ki67 proliferative index of 30–35% (Fig. 1J). The final pathology was a glioblastoma multiforme (GBM, WHO Grade IV).

### *PNKP* mutation analysis

Following institutional review board (IRB) approval and parental consent, we profiled blood samples from the patient and both parents. Using a sequencing panel for microcephaly, the blood-derived patient and parental DNA were sent for sequencing at the University of Chicago Genetic Services, Chicago, Illinois (https://dnatesting.uchicago.edu/tests/microcephaly-panel). The results revealed two changes in the *PNKP* gene. The first change, NM_007254.3:c.968C>T, abbreviated as 968C>T, converts a highly conserved threonine to methionine (T323M) in the functional domain of PNKP. The second change, NM_007254.3:c.302C>T, abbreviated 302C>T, converts a moderately conserved amino acid in the FHA domain (P101L) that has not been previously reported. Therefore, this mutation was of unknown significance. Mutational analysis of his parents, however, showed that they both carried the 302C>T mutation. Further sequencing analysis of the tumor sample revealed several molecular alterations, including deletion of α thalassemia/mental retardation syndrome X-linked (*ATRX*), pathogenic mutations with corresponding loss of heterozygosity in tumor protein 53 (*TP53*) and Neurofibromatosis 1 (*NF1*), single copy deletion of breast cancer 2 (*BRCA2*) and retinoblastoma 1 (*RB1*), and amplification of cyclin dependent kinase 4 (*CDK4*).

### *PNKP* mutants have weaker DNA kinase and phosphatase activities

**Figure 2:**
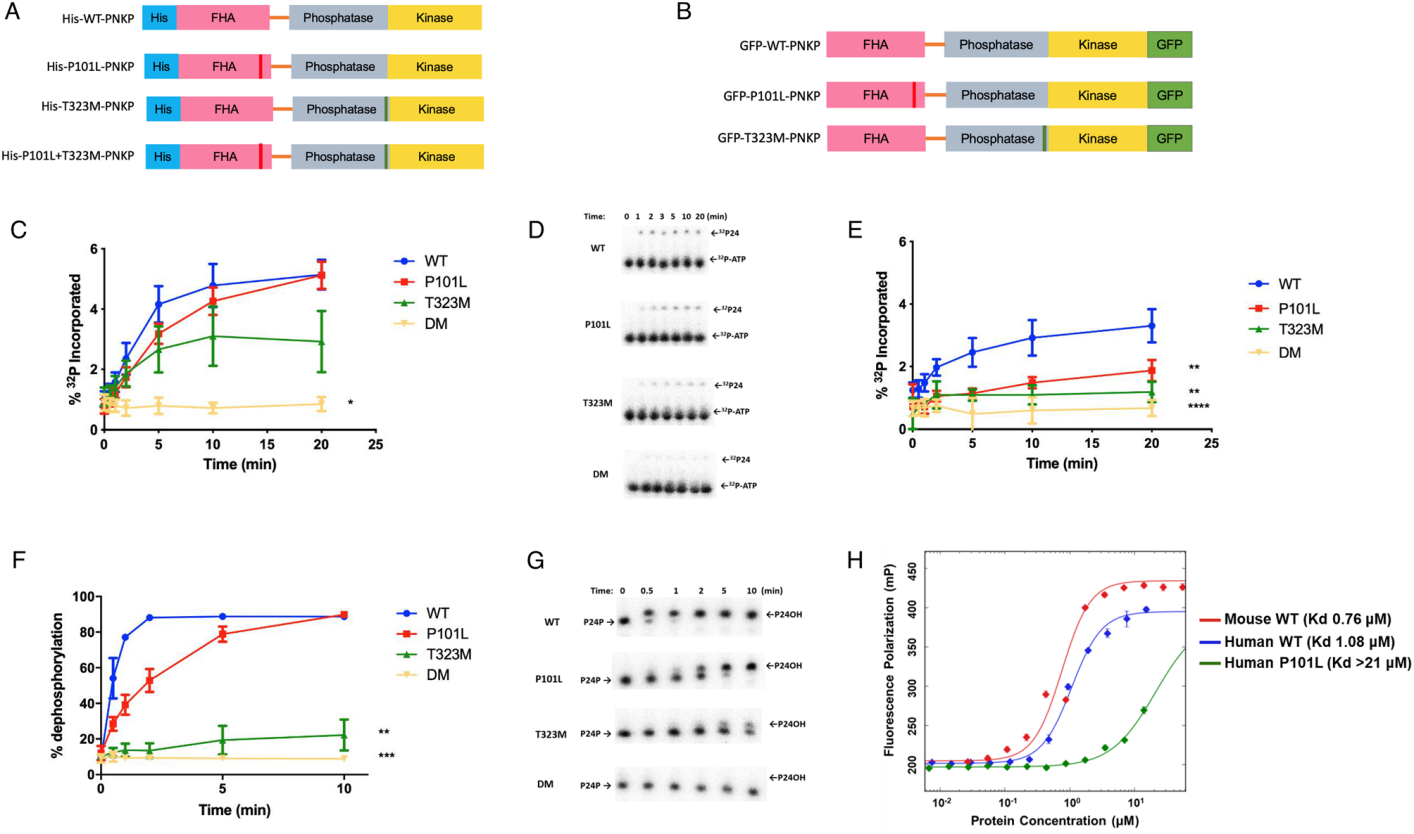
Measurement of 5′-kinase and 3′-phosphatase activities of wild-type and mutant PNKPs. (**A**,**B**) The structures of mutant PNKP constructs designed to monitor the effects of the PNKP mutations found in the MCSZ patient. FHA represents the forkhead-associated domain of PNKP. His-indicated histidine tag (**A**) and GFP indicates the green fluorescent protein tag (**B**). (**C**) Kinase assay: a 24mer oligonucleotide bearing a 5′-hydroxyl terminus was incubated with wild-type or P101L, T323M, and the double mutant (DM) variant PNKP proteins in the presence of a tenfold molar excess of radiolabelled ATP and then analyzed by gel electrophoresis. (**D**) Representative gel image of PNKP kinase assay. ^32^P24: 24-mer oligonucleotide substrate bearing a ^32^P-label at the 5′-terminus. ^32^P-ATP: ^32^P-labelled ATP ([γ-^32^P] ATP). (**E**) To examine the temperature-dependent destabilization of wild-type and mutant forms of PNKP, the proteins were preincubated at 37 °C before carrying out the kinase assay. (**F**) Phosphatase assay: a 5′-labelled 24-mer oligonucleotide bearing a 3′-phosphate group was incubated with wild-type or variant PNKP protein and then analyzed by gel electrophoresis. (**G**) Representative gel image of PNKP phosphatase assay. P24P: 24-mer oligonucleotide substrate bearing a ^32^P-label at the 5′-terminus as well as a 3′-phosphate. P24OH: 24-mer oligonucleotide substrate bearing a ^32^P-label at the 5′-terminus, and 3′-hydroxyl. (**H**) Interaction of wild-type and P101L PNKP with an XRCC4-based peptide. The interaction was monitored by fluorescence polarization of the fluorescent-dye labeled XRCC4-based phosphopeptide (GGYDES-pT-DEESKK) in the presence of increasing concentrations of wild-type murine and human wild-type or P101L PNKP. In each case the data represent the mean ± SEM of three independent experiments. Ordinary one-way ANOVA followed by Tukey’s multiple comparisons test was performed using GraphPad Prism 7.0, GraphPad Software. *p < 0.05, **p < 0.01, ****p < 0.0001.

For analysis of the biochemical consequences of the *PNKP* mutations we expressed and purified the P101L and T323M proteins arising from the 302C>T and 968C>T mutated cDNA, respectively (Fig. 2A,B). We also generated a double-mutant (PNKP-DM) containing both the P101L and T323M altered amino acids (Fig. 2A,B). The T323M mutant displayed a markedly reduced kinase activity, while the activity of the P101L mutant was only slightly lower than the wild-type protein (Fig. 2C,D). In contrast, PNKP-DM completely lost activity. In the case of the T323M mutant, it was noticeable that although it exhibited significant activity at the start of the reaction, the activity plateaued after 10 min only achieving ~ 50% reaction (Fig. 2C). One possibility for this is protein instability. This possibility was examined by pre-incubation of the protein at 37 °C for 10 min prior to addition of the substrate and adenosine triphosphate (ATP; Fig. 2E). Although the wild-type and P101L proteins retained residual activity, the T323M mutant exhibited almost complete heat inactivation. An examination of the phosphatase activity revealed that the P101L mutant retained significant activity albeit slower than the wild-type protein (Fig. 2F,G). In contrast, the activity of the T323M protein was severely curtailed, while PNKP-DM exhibited almost no phosphatase activity (Fig. 2F,G).

### Decreased binding affinity between mutant PNKP and DNA substrates

To determine if reduced enzyme activity was due to lower substrate binding, we examined the affinities (*K*_*d*_) of the mutant enzymes towards two double-stranded substrates containing a single-nucleotide gap with strand break termini reflecting the need for kinase (5′-OH) and both kinase and phosphatase (5′-OH and 3′-P) activity using steady-state fluorescence. The binding affinities between P101L PNKP and the DNA substrates does not differ markedly from those of the wild-type protein (Supplemental Table S1). This is expected since P101L still retains relatively strong kinase and phosphatase activities. In contrast and consistent with their respective enzymatic activity, the T323M and PNKP-DM showed significantly reduced binding affinities (Supplemental Table S1). Previous studies have indicated that the DNA-binding surfaces of PNKP reside in the catalytic domain^24^. It is therefore reasonable that the P101L mutation in the FHA domain would not substantially change the DNA binding affinity. The crystal structure of PNKP revealed that there is an intimate association between the phosphatase and kinase subdomains^17^, therefore, it is not surprising that the T323M mutation, although located within the phosphatase domain, can reduce the binding affinity for the 5′-OH substrate (GAP1). Analysis of the protein structure by circular dichroism, however, did not reveal a gross deformation caused by the T323M mutation (Supplemental Fig. S1 and Supplemental Table S2). Since the P101L mutation lies in the FHA domain, which interacts with the phosphorylated scaffold proteins XRCC1 and XRCC4, we employed fluorescence polarization to examine the binding of the wild-type and mutant PNKP protein to a peptide sequence based on XRCC4 that contains the key phospho-threonine residue^17^. The results indicate that the P101L alteration causes a significantly reduced binding affinity to the phosphopeptide compared to the wild-type PNKP (Fig. 2H).

### Influence of PNKP mutation on cellular protein levels

Low cellular levels of PNKP appear to be a common feature of mutants associated with cases of MCSZ^9,12^. Due to the unavailability of live cells from the patient, the influence of the P101L and T323M mutations was investigated at the cellular level using PNKP knock-out HeLa cells (HeLa PNKP^−/−^)^25^ transfected with vectors encoding either the wild-type or mutant GFP-tagged PNKP proteins. Western blot analysis following transient transfection revealed that the cellular levels of the mutant proteins were lower than those of the transfected wild-type PNKP, ~ 50% in the case of the P101L mutant and only ~ 10% for the T323M mutant (Supplemental Fig. S2A and B). We therefore asked if the reduced protein levels arose from reduced transcription or if the reduction was purely at the protein level. Transcription of the cDNAs with the 302C>T and 968C>T mutations was ~ 50% and ~ 60% lower, respectively, than the wild-type cDNA (Supplemental Fig. S2C). Thus the lower level of the P101L protein could be accounted for by reduced transcription, but although there was a marked reduction in the transcription of the 968C>T cDNA, this cannot fully explain the low level of T323M-PNKP observed in the western blot, suggesting that either the translation process producing T323M-PNKP is less efficient, or that T323M-PNKP protein is less stable. Therefore, to overcome the disparity of PNKP expression in transiently transfected cells, we established and utilized clonally-derived stably transfected cell lines expressing similar levels of wild-type and mutant PNKP (Supplemental Fig. S3).

### Influence of *PNKP* mutation on protein localization

**Figure 3:**
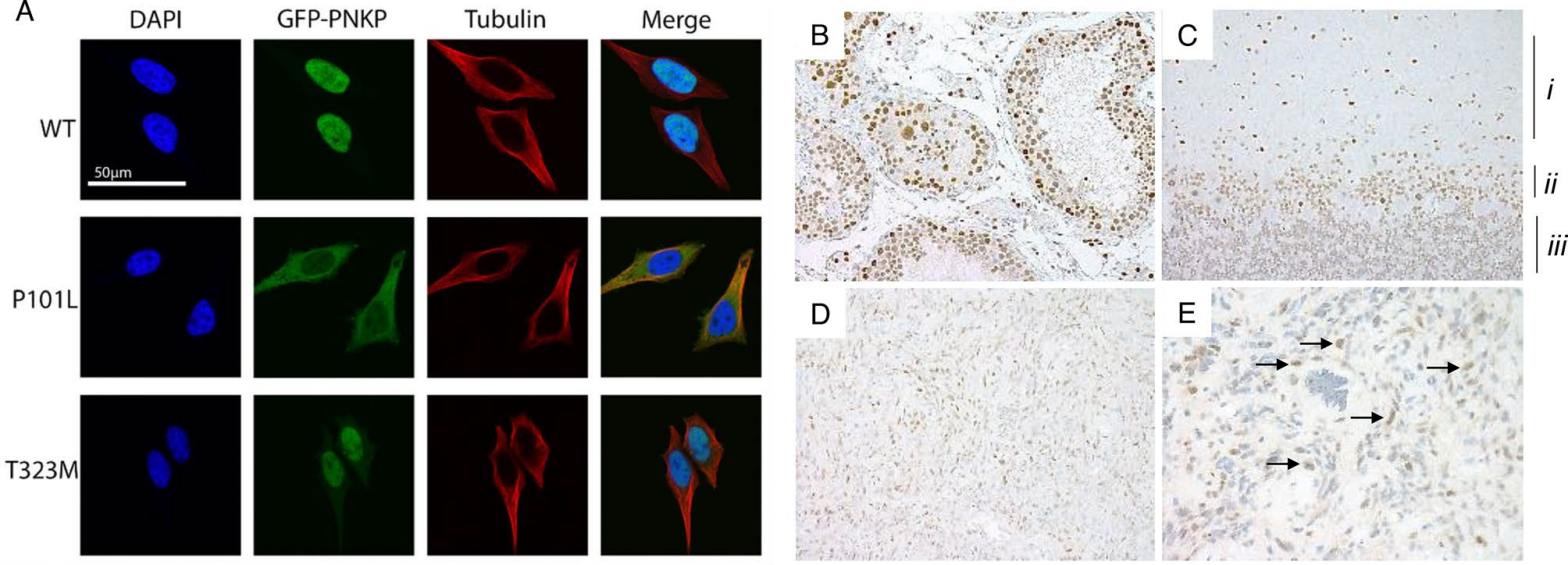
Cellular localization of PNKP. (**A**) HeLa PNKP^−/−^ cells were stably transfected with indicated GFP-tagged PNKP constructs (green), tubulin immunofluorescence (red) indicates the cytoplasmic area, Hoechst staining (blue) indicates the nuclear area. Images were captured using an LSM710 confocal microscope. (**B**,**C**) PNKP protein expression is restricted to the nucleus in normal tissue (b—Testes, c—Cerebellum. I—Molecular layer, ii—Purkinje layer, iii—granular layer). (**D**,**E**) PNKP expression in the patient derived tumor tissue is nuclear and cytoplasmic (higher magnification image with back arrows in **E**).

Next, we examined the cellular localization of the mutant PNKP proteins following transfection of cDNA for GFP-tagged PNKP. The wild-type PNKP stably re-expressed in HeLa PNKP^−/−^ cells predominantly localized to the nucleus (Fig. 3A). Similarly, the T323M PNKP-GFP mutant localized to the nucleus, although these cells appeared to have a slightly higher cytoplasmic signal relative to wild-type PNKP (Fig. 3A). The P101L PNKP-GFP mutant, however, was predominantly cytoplasmic (Fig. 3A). A western blot analysis verified that P101L PNKP-GFP maintained its full length with no degradation (Supplemental Fig. S3), implying that the cytoplasmic signal arose from the PNKP-GFP full length protein rather than a GFP-tagged cleavage fragment. These localization patterns were observed in both transiently and stably transfected cells. To quantify the distribution pattern and exclude the possible bias from the selection of stably-transfected cell lines, we used high content screening to analyze over 2000 transiently-transfected cells in each group. The ratio between the area of the GFP signal and the area of the nucleus (Hoechst) within each cell was determined and it confirmed that the P101L mutation significantly changed the PNKP distribution pattern, with most cells having a GFP/Hoechst area ratio larger than 2 (Supplemental Fig. S4). Similar to wild-type HeLa cells, wild-type PNKP is restricted to the nucleus in normal human testes and cerebellum (Fig. 3B,C). Consistent with the localization of mutant PNKP-GFP in the HeLa PNKP^−/−^ cells, the patient’s tumor showed both nuclear and aberrant cytoplasmic distribution of PNKP (Fig. 3D,E).

**Figure 4:**
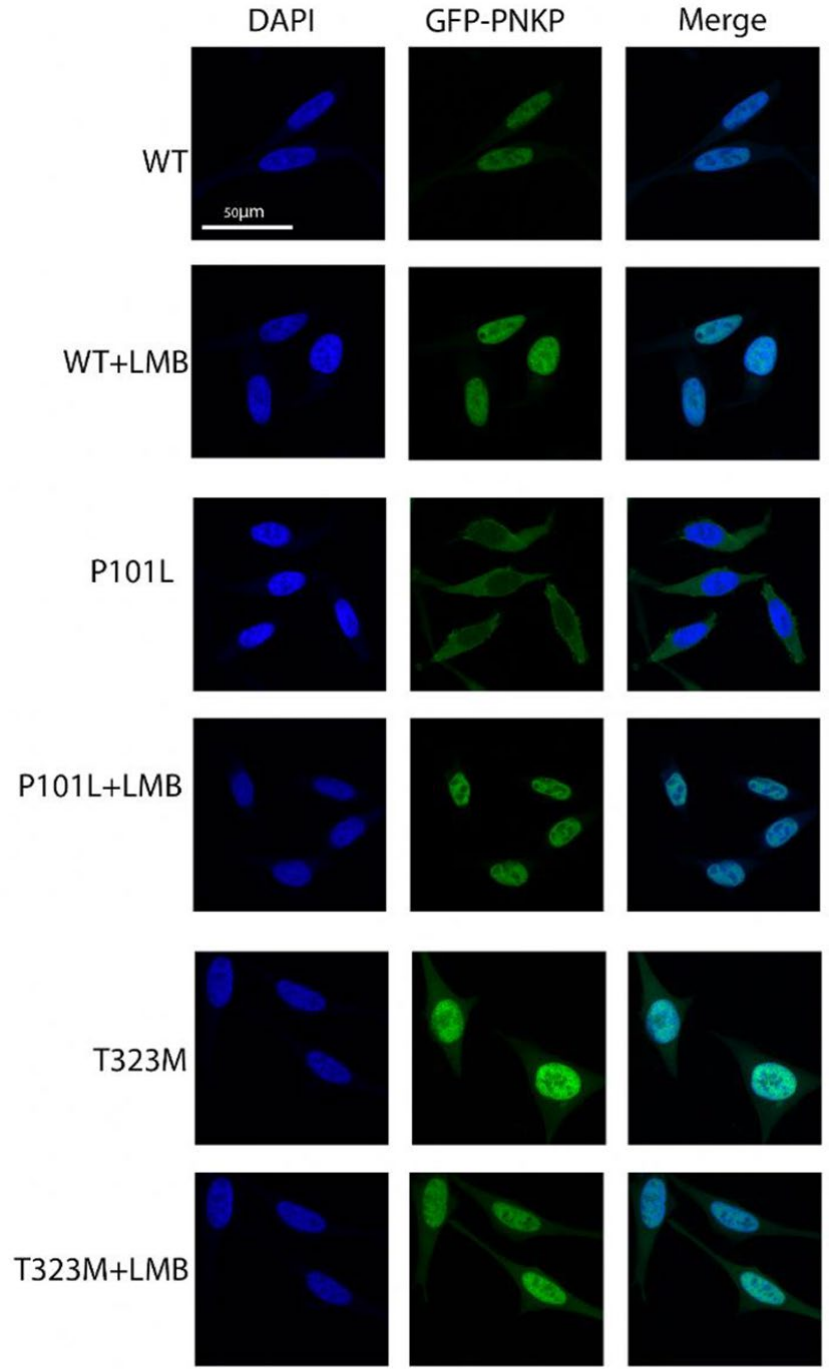
Inhibition of exportin 1 leads to nuclear retention of P101L PNKP. Stably-transfected PNKP-knockout HeLa cells expressing GFP-tagged wild-type and mutant PNKP were treated with leptomycin B (LMB) for 3 h and then stained with DAPI and imaged at 40 × magnification.

The transportation of protein through the nuclear membrane is bi-directional. Previously our group discovered a bipartite nuclear localization signal (NLS) located in the flexible linker between the FHA domain and catalytic domain (K130, R131 + KKRMRK, 137–142)^26^, which has recently been confirmed by others^27^. While the P101L may potentially impact the NLS, another possible reason for the altered localization pattern is that the mutation created a novel nuclear export signal (NES) in PNKP. The NES signal is a leucine-rich peptide region first discovered in cAMP-dependent protein kinase inhibitor (PKI)^28^ and human immunodeficiency virus type 1 (HTV-1) Rev protein^29^. Export protein exportin 1 (CRM1) has been identified as the export receptor for proteins that harbor an NES signal and transports them from the nucleus to the cytoplasm^29–32^. We used two different NES prediction software programs to analyze the wild-type and mutant PNKP protein sequence. Both NetNES^33^ and LocNES^34^ indicate the P101L mutation creates a new NES signal in the FHA domain while T323M does not create any NES signal (Supplemental Table S3). To confirm this prediction, we monitored the influence of leptomycin B (LMB), an inhibitor of exportin 1^31,35^ on PNKP localization. The results showed LMB treatment significantly changed the localization pattern of P101L PNKP with nearly all PNKP-GFP being retained in the nucleus (Fig. 4).

### Influence of PNKP mutations on radiation sensitivity

**Figure 5:**
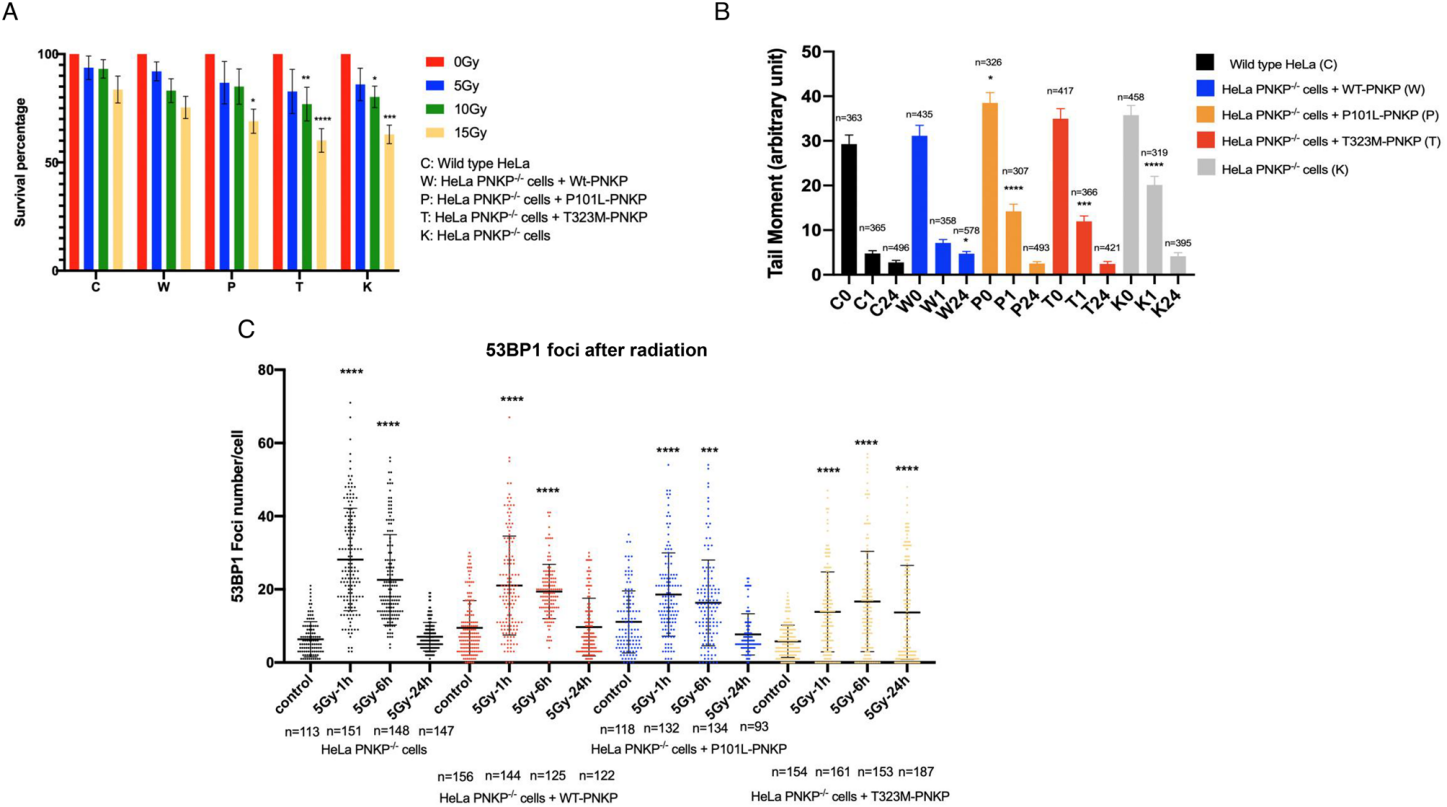
Influence of PNKP variants on cellular response to radiation and DNA repair. (**A**) Viability of wild-type and mutant cell lines at different radiation dosages. Plots represent mean $\pm \hspace{0.17em}$SD. Ordinary one-way ANOVA was performed to compare each cell line with the wild-type HeLa at the same radiation dose using GraphPad Prism 7.0. (**B**) Single-strand break repair measured by the single cell gel electrophoresis assay. DNA tail moments of cell lines were measured at 1 and 24 h after irradiation. Each column includes data from at least 300 cells. Plots represent mean ± SEM. At each time point, one-way ANOVA was performed to compare each group with the wild-type un-transfected HeLa cells at the same time point using GraphPad Prism 7.0. (**C**) Repair of DNA double strand breaks by the NHEJ pathway based on 53BP1 foci numbers observed in the wild-type and stably transfected cell lines at different times after irradiation, *n* indicates the measured cell numbers in each group. The plots represent mean ± SD. In each graph, one-way ANOVA was performed to compare 53BP1 foci at each time point with the number of foci in unirradiated cells using GraphPad Prism 7.0, GraphPad Software. ***p < 0.001, ****p < 0.0001.

Hydroxyl radicals produced endogenously or by ionizing radiation generate strand break termini containing a high percentage of 3′-phosphate and to a lesser extent 5′-OH termini^36,37^. To carry out an examination of the influence of the PNKP mutations on cellular response to oxidative DNA damage and repair, the wild-type and stably transfected HeLa PNKP^−/−^ cells were subjected to increasing doses of γ radiation. The un-transfected wild-type HeLa cells showed the most radiation-resistant phenotype, while the HeLa PNKP^−/−^ cells displayed the greatest radio-sensitivity, similar to our previous observations^16^ (Fig. 5A). The re-expression of wild-type PNKP in HeLa PNKP^−/−^ cells re-established resistance to radiation. The P101L mutant cells exhibited slightly increased radio-sensitivity, while the T323M mutant cells showed similar radio-sensitivity to the knockout cells (Fig. 5A).

### Influence of PNKP mutations on ionizing radiation-induced DNA repair

To further examine the relevance of mutant PNKP after radiation, we monitored the capacity of the cells to repair radiation-induced DNA damage. The alkaline single cell gel electrophoresis (comet) assay primarily detects DNA single-strand breaks and alkali-labile sites in the DNA^38,39^. All cell lines repaired most of the DNA damage within 24 h (Fig. 5B). However, a significant difference was observed at early times (1 h), with the HeLa wild-type cells and the HeLa PNKP^−/−^ cells transfected with wild-type PNKP both displaying a rapid reduction in tail moment (indicating efficient repair), while HeLa PNKP^−/−^ cells showed the slowest repair progress (Fig. 5B). The two mutant cell lines displayed intermediate repair efficiency (Fig. 5B).

PNKP participates in the non-homologous end joining pathway (NHEJ) but is not involved in the homologous recombination (HR) pathway^40^, therefore to determine the influence of mutant PNKP in NHEJ repair we monitored the formation and disappearance of 53BP1 foci^41^ following cell irradiation. HeLa PNKP^−/−^ cell lines exhibited the highest number of foci at 1- and 4-h post-radiation but returned to background levels by 24 h (Fig. 5C). This is similar to our previous observations monitoring the γH2AX signal (another marker of double-strand breaks) in PNKP knockdown A549 lung cancer cells^42^. A recent paper reported the existence of a yet unidentified alternative 3′-phosphatase that can act at DSB^35^, which may explain the eventual repair of radiation-induced DSB in HeLa PNKP^−/−^ cells. The cell lines complemented with wild-type and P101L mutant displayed similar foci levels at each time point, indicating that the P101L expressing cells repair double-strand breaks with near normal kinetics and that sufficient PNKP is retained in the nucleus to carry out the repair. In contrast, cells expressing the T323M PNKP failed to return to background level by 24 h (Fig. 5C). This suggests that mutant T323M PNKP is inefficient at repairing DSB, likely due to a combination of its intrinsically poor enzymatic capacity and by impeding access of the alternative, yet to be identified, backup repair enzyme(s) to the damaged termini^25^.

### Anchorage-independent growth raised in mutant cell lines

Anchorage-independent growth is frequently used as an indicator of pro-oncogenic transformation^43^. The soft agar colony formation assay is a well-established method for characterizing anchorage-independent growth capacity and is one of the most stringent tests for malignant transformation in cells^44^. The higher concentration of agar in the growth environment prevents cells from adhering yet allows transformed cells to form visible colonies^45^. HeLa PNKP^−/−^ cells demonstrated increased transformation frequency approximately fourfold over wild-type HeLa cells (Supplemental Fig. S5). HeLa PNKP^−/−^ cells transfected with wild-type PNKP reduced the number of transformants similar to the parental HeLa cell line. Expression of the P101L and T323M mutant PNKP in HeLa PNKP^−/−^ cells, however, increased the transformation frequency two- to threefold (Supplemental Fig. S5).

## Discussion

Several DNA repair disorders, such as Ataxia telangiectasia, are known to be associated with both neurological dysfunction and elevated cancer risk. MCSZ is an extremely rare autosomal recessive disorder and to date there has been no indication of elevated cancer risk associated with MCSZ, although one case of a lower-grade cerebellar pilocytic astrocytoma was diagnosed in a patient with AOA4^4^. Since the acceptance of this paper, another manuscript^46^ was published describing another pediatric patient with MSCZ who developed acute myeloid leukemia. This adds to the growing body of evidence linking PNKP with cancer. The occurrence of primary CNS tumors in children in the US is ~ 5.3 cases per 100,000 children, of which the high-grade brain tumor, glioblastoma multiforme (GBM) accounts for 3–15%^47,48^. Although not definitive, these cases of relatively rare brain tumors in AOA4 and MCSZ strongly suggest a link between *PNKP* mutation and elevated cancer risk. However, since complete loss of PNKP is likely to be embryonic lethal^49^, some residual activity is required for survival^50^ and so it is important to characterize the mutant proteins in terms of their enzyme activity and cellular impact.

**Figure 6:**
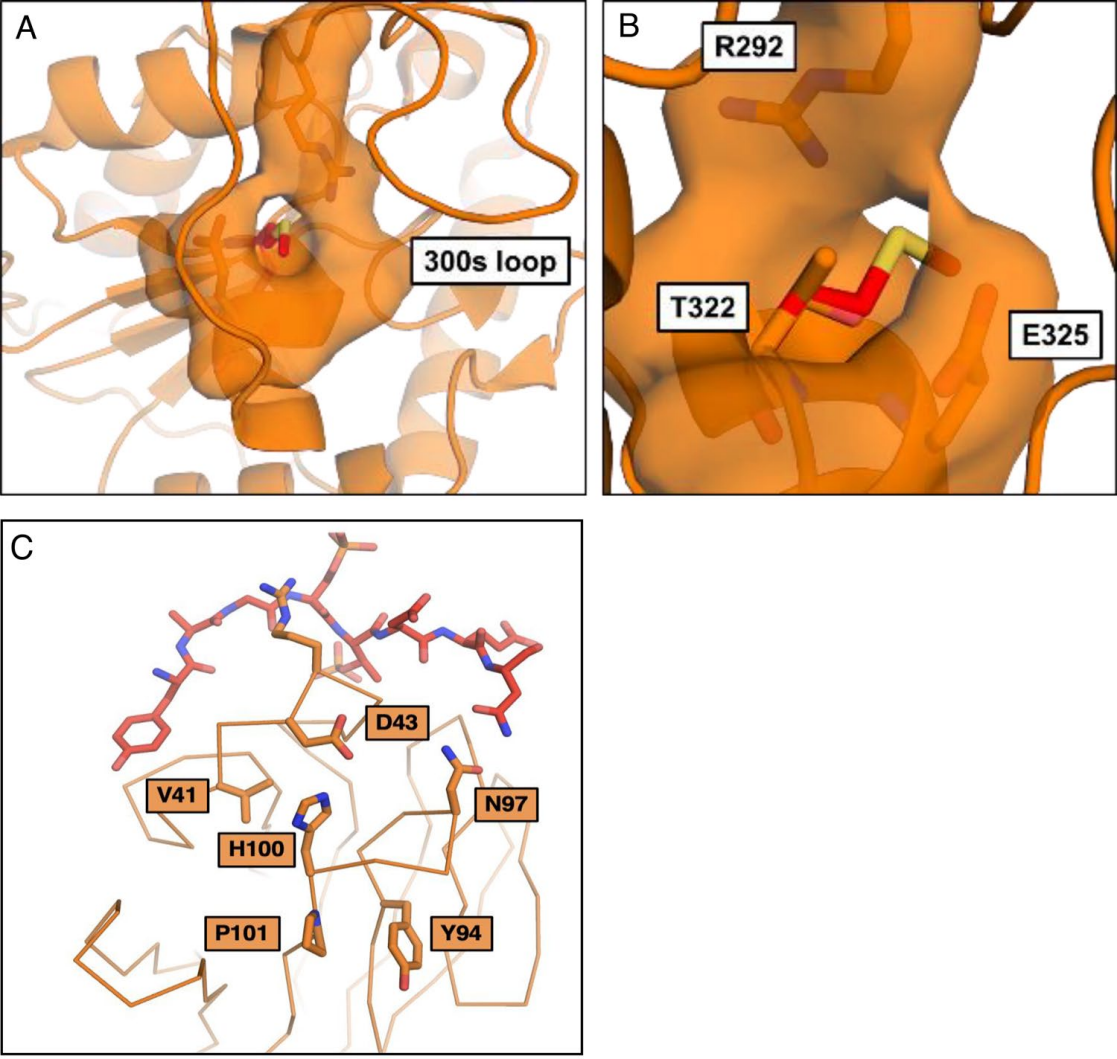
Local structures within PNKP at the sites of the amino acid changes. (**A**,**B**) Images taken from murine PNKP surrounding T322 (T323 in human PNKP). The mutation T322M appears to be minimally disruptive despite the larger size of methionine, due to the presence of a pocket that can accommodate the extended amino acid. However, potential clashes can occur with Glu 325 and Arg 292 (Glu 326 and Arg 293 in human PNKP). (**C**) An alignment of the FHA domain of human PNKP with the phosphopeptide binding site of XRCC4. The PNKP FHA domain is shown in orange and the XRCC4 pThr/pSer peptide is shown in red (PDB: 2W3O). A disruption in the P101/Y94 loop that causes a conformational change will affect His 100 which would affect the pThr binding interface through packing with V41 and D43. Additionally, movement in the P101/Y94 loop would affect direct binding to the pThr peptide through N97’s hydrogen bonds.

Based on the location of the mutation, the P101L alteration did not significantly affect either the kinase or phosphatase activities since the altered amino acid residue lies in the FHA domain rather than the catalytic domain. In contrast, the T323M alteration severely curtailed the PNKP phosphatase activity and to a lesser extent the kinase activity. Studies on the biochemical and cellular consequences of PNKP mutation causing MCSZ are limited. Understandably, substantial structure loss such as frameshifts, e.g. T424Gfs, could reduce protein stability and enzymatic activities. But it is interesting that single mutations, such as L176F, also showed reduced enzymatic activities^21^. Both mutations found in our patient are one amino acid replacements. The purified mutant PNKPs retained their full size and the circular dichroism analysis suggested that the structure of the more affected T323M mutant did not appear to be grossly altered by the change in amino acid despite the larger size of methionine compared to threonine. This is probably due to a pocket that the extended amino acid can fit into with relatively minor clashes predicted with Glu 326 and Arg 293. We show the structure for the highly conserved 300s loop within mouse PNKP^17^ in which the equivalent amino acids are Glu 325 and Arg 292 (Fig. 6A,B). However, the mutation to methionine does remove a hydrogen bond between the main chain nitrogen of Glu 326 and the hydroxyl of Thr 323. The clashes with Glu 326 and Arg 293, along with the missing hydrogen bond, could disrupt the loop between residues 291 and 307 (300s loop), which has been shown to be important in binding double stranded DNA substrates^24^ which in turn may explain the reduced affinity of the T323M mutant for the double stranded DNA substrates (Supplemental Table S1). The fact that this mutation appears minimally disruptive and yet causes such a pronounced phenotype speaks to the necessity and sensitivity of the PNKP phosphatase domain.

While the P101L mutation did not significantly affect its affinity for DNA substrates or its enzymatic activity, it did reduce its affinity for the XRCC4-based phosphopeptide. This reduction in affinity is likely due to conformational changes caused by the mutation and resulting clashes with Y94. A conformational change in the P101/Y94 loop will likely affect the pThr binding interface through residues H100 and N97 (Fig. 6C). However, the degree of reduced binding to the phosphopeptide may not entirely reflect reduced binding to full-length phosphorylated XRCC4 since other protein–protein interactions are involved in the binding between the two full-length proteins and there is also an interaction between the two proteins that is not dependent on XRCC4 phosphorylation^51^. It is also noticeable that DSB repair, as judged by 53BP1 foci, was not significantly different in the cells expressing the P101L mutant protein compared to cells complemented with the wild-type protein. An unanticipated consequence, however, of the novel P101L mutation is the alteration of its cellular localization. To date there are no other PNKP mutations that have been reported to contribute to localization changes. Similar to the result we observed with the cells expressing mutant P101L PNKP-GFP, the IHC result of the patient showed cytoplasmic localization of PNKP. The NES analysis and LMB treatment indicate that the P101L mutation creates a new nuclear export signal that enables binding to exportin 1 and export into the cytoplasm. Although it is not uncommon for disease-associated mutations to alter the cellular localization of proteins, to our knowledge there is only one other report of a gain-of-function mutation that results in the formation of a new NES^52^. In the latter case a mutation in the nucleophosmin gene, *NPM*, linked to acute myeloid leukemia was found to generate an additional NES responsible for relocalizing NPM to the cytoplasm.

Expression of mutant PNKP proteins revealed several consequences that together raise the possibility that PNKP mutations could lead to tumorigenesis as well as MCSZ. As seen with other PNKP mutations, the level of the mutant proteins, particularly the T323M PNKP, was significantly depressed. Taking this into account together with its severely curtailed level of enzymatic activity would imply that this variant of the protein will provide extremely limited DNA repair capacity. Indeed, even in cells expressing artificially high levels of the T323M variant the repair of radiation-induced double-strand breaks is far from complete after 24 h (Fig. 5C). Another important consequence of defective DNA repair is increased spontaneous mutation frequency. Spontaneous mutations continually arise from endogenous genotoxic agents in live cells such as ROS^53^. We have previously shown that shRNA-mediated knockdown of PNKP in human A549 lung cancer cells led to a sevenfold increase in the spontaneous mutation frequency^42^. Sequence analysis of the proband’s tumor sample showed that it carried multiple molecular alterations in addition to the *PNKP* mutations, including deletion of *ATRX*, mutations with corresponding loss of heterozygosity in *TP53* and *NF1*, copy loss of *BRCA2* and *RB1*, and amplification of *CDK4*. *TP53* and *ATRX* mutations are commonly found in pediatric GBM^54^, and a recent study revealed that inactive ATRX in Trp53 deficient murine neuroepithelial progenitors (mNPCs) altered the transcriptional patterns strongly correlated with several glioma signatures^55^. Through the mutual exclusivity analysis of all listed studies on cBioPortal (including data from the cancer genome atlas; TCGA), all 6 genes mentioned above showed significant co-occurrence tendency with *PNKP* mutations (Table 1). Since the entire genome of the patient was not sequenced, the mutation time of different genes are difficult to determine. However, it is possible that in our patient, mutant PNKP induced impairment in DNA damage repair, preceded and synergized with pediatric glioma associated mutations such as ATRX and TP53 resulting in brain tumor initiation and progression.

**Table 1:** Mutual exclusivity analysis of PNKP and candidate mutations that were found in the patient’s tumor sample.

A	B	Neither	A not B	B not A	Both	Log2 Odds ratio	p value	q value	Tendency
PNKP	BRCA2	9520	97	546	28	2.331	< 0.001	< 0.001	Co-occurrence
PNKP	ATRX	9394	101	672	24	1.732	< 0.001	< 0.001	Co-occurrence
PNKP	NF1	9274	102	792	23	1.401	< 0.001	< 0.001	Co-occurrence
PNKP	RB1	9320	103	746	22	1.416	< 0.001	< 0.001	Co-occurrence
PNKP	TP53	6258	63	3808	62	0.694	0.005	0.007	Co-occurrence
PNKP	CDK4	9787	120	279	5	0.548	0.269	0.283	Co-occurrence

Mutual exclusivity between PNKP and candidate mutations in TCGA PanCancer Atlas studies in cBioPortal. Neither: Numbers of samples with alterations in neither A or B. Only A/Only B: Numbers of samples with alterations in only gene A/B. Both A + B: Numbers of samples with alterations in both gene A and B. Log2 odds ratio: Quantifies how strongly the presence or absence of alterations in A are associated with the presence or absence of alterations in B in the selected samples. OR = (Neither * Both)/(A Not B * B Not A). Log2 odds ratio > 0: Tendency towards co-occurrence. Log2 odds ratio < = 0: Tendency towards mutual exclusivity. p-value: derived from one-side Fisher Exact Test. q-Value: derived from Benjamini–Hochberg FDR correction procedure.

In conclusion, although functional studies in a mouse model are needed to characterize the influence of both the novel P101L and previously described T323M PNKP alterations on brain tumor initiation, we speculate that mutant PNKP-driven impaired DNA damage response and higher spontaneous mutation rates contributed to the generation of pediatric glioma associated driver mutations such as TP53 and ATRX in the clinical case described.

## Material and methods

### Tumor sample template preparation, gene capture and massively parallel sequencing

Tumor DNA sequencing was preformed using the University of Washington UW-OncoPlex version 5, a clinically validated method as previously reported (https://testguide.labmed.uw.edu/public/view/OPX)^56^. Briefly, after DNA extraction, sequencing libraries were prepared using KAPA HyperPrep (Roche, Wilmington, MA) and hybridized to a custom set of complementary RNA (cRNA) biotinylated oligonucleotides targeting the exons of 262 genes and select intronic regions for a total of ~ 2 Mb of targeted DNA sequenced. Next generation sequencing (NGS) was performed using a NextSeq500 sequencing system (Illumina, San Diego, CA) and data analysis was performed using custom bioinformatics developed by the UW NGS Analytics Laboratory.

### Expression plasmids and site-directed mutagenesis

For the production and purification of PNKP protein, pET-16b (Novagen Inc., Madison, WI) bacterial expression plasmid harboring the full-length human *PNKP* cDNA was generated following previously reported procedures^15,57^. To generate fluorescently tagged wild-type and mutant PNKP proteins in mammalian cells, the full-length cDNA was subcloned into the pCMV6-AC-mGFP (Origene, Rockville, MD) mammalian expression plasmid as described before^58^. To generate the desired *PNKP* single point mutants (C302T and C968T), the QuickChange II site-directed mutagenesis kit (Stratagene, La Jolla, CA) was used, following the manufacturer’s protocol and using the mutagenic primers shown in Supplemental Table S4. The *PNKP* double mutant (DM) was generated by using the C968T primers with the C302T-mutated cDNA in a similar procedure. Finally, the mutants were sequence validated by the Applied Genomics Core at the University of Alberta. Besides the site directed mutagenesis sites (C302T for P101L mutation; C968T for T323M mutation), the PNKP and GFP sequences among all constructs are full length and identical. However, we found a small difference in the linker that connects PNKP and GFP: the WT-PNKP codes for 4 more amino acids in the linker than P101L and T323M-PNKP constructs, the linkers between P101L and T323M PNKP are identical. This explains the slightly shorter size of the P101L and T323M proteins seen on western blots (Fig. S2A). However, it is important to note that this change in the linker would not affect PNKP enzymatic activity or localization or change our interpretation of the results.

### Expression and purification of mutant PNKPs

The PNKP wild-type and mutant bacterial expression plasmids were transfected into *E. coli* bacterial strain BL21 (DE3) (NEB). The bacteria were grown at 37 °C in 4 L of lysogeny broth (LB) containing ampicillin (50 µg/mL) to reach an OD_600_ of 0.6. Protein expression was then induced by overnight incubation at 18 °C in the presence of 100 µM isopropyl-β-d-1-thiogalactopyranoside (IPTG, Sigma, St. Louis, MO). After induction, the bacteria were harvested by centrifugation at 10,000*g* for 10 min at 4 °C and resuspended in 50 mL of lysis buffer (150 mM NaCl, 50 mM Tris–HCl, pH 8.0, 1 mM ethylenediaminetetraacetic acid (EDTA), 0.1% β-mercaptoethanol, 0.5 mM phenylmethylsulfonyl fluoride (PMSF), 0.5 mg/mL lysozyme). After stirring on ice for 30 min, the bacteria were disrupted by sonication. The soluble fraction was separated by centrifugation at 15,000*g* for 30 min at 4 °C. To precipitate nucleic acids, 10% polyethyleneimine was added dropwise to the soluble fraction to a final concentration of 0.3%, the sample was then stirred on ice for 20 min and centrifuged at 15,000*g* for 20 min at 4 °C. Protein in the supernatant was then precipitated by addition of ammonium sulfate to a final concentration of 50%, and then centrifuged at 15,000*g* for 30 min at 4 °C. The protein pellet was resuspended in solution and purified through a HiPrep 16/10 Butyl FF column (Amersham Pharmacia BioTech, Baie d’Urfe, PQ), SP Sepharose Fast Flow cation-exchange column (Amersham Pharmacia BioTech, Amersham, UK) and HiLoad 16/60 Superdex 75 gel filtration column (Amersham Pharmacia BioTech) as described before^57^. The purity and integrity of the protein were confirmed by gel electrophoresis and Coomassie Brilliant Blue staining (Supplemental Fig. S6).

### PNKP kinase assay

The 5′-kinase activities of wild-type and mutant PNKP proteins were measured by a kinase assay modified from procedures described before^15^. Briefly, PNKP (500 ng) was added to a reaction mixture (40 µL total volume) containing kinase buffer (80 mM succinic acid, 10 mM MgCl_2_ and 1 mM DTT, pH 5.5), 0.5 mM 24-mer oligonucleotide substrate (5′-GGCGCCCACCACCACTAGCTGGCC-3′) with 5′-OH termini, 5 mM unlabeled ATP, and 5 µCi of [γ-^32^P] ATP (3000 Ci/mmol, Amersham Pharmacia Biotech). The reaction mixture was incubated at 37 °C for 0.5, 1, 2, 5, 10 and 20 min. 5 µL of the sample was mixed with 2.5 µL of 3 × sequencing gel loading dye (Fisher Scientific, Edmonton, AB), boiled for 10 min to stop the reactions then run on a 12% polyacrylamide gel containing 7 M urea at 200 V for 30 min. The gel was scanned on a Typhoon 9400 Variable mode imager (GE Healthcare Life Sciences, Mississauga, ON) and quantified using ImageQuant 5.2 (GE Healthcare Life Sciences). In the experiment to test the stability of the mutant proteins in vitro, all the purified wild-type/mutant PNKP proteins were pre-heated at 37 °C for 10 min before being added into the reaction mixture.

### PNKP phosphatase assay

The 3′-phosphatase activities of the wild-type and mutant PNKP proteins were measured by a phosphatase assay modified from previous studies^42,59^. Briefly, PNKP (50 ng) was added to a reaction mixture (20 µL total volume) containing phosphatase buffer (70 mM Tris–HCl, 10 mM MgCl_2_, 5 mM DTT, pH 7.6), 4 µM 24-mer oligonucleotide substrate (5′-GGCGCCCACCACCACTAGCTGGCC-3′) bearing a ^32^P-label at the 5′-terminus as well as a 3′-phosphate. The reaction was carried out at 37 °C for 0.5, 1, 2, 5 and 10 min. 3 µL of the sample was mixed with 1.5 µL of 3 × sequencing gel loading dye, boiled for 10 min to stop the reaction then run on a 12% polyacrylamide gel containing 7 M urea at 1800 V for 3 h. The gel was then scanned on a Typhoon 9400 Variable mode imager and quantified using ImageQuant 5.2.

### Steady-state fluorescence spectra study

The affinity of mutant PNKP protein for DNA substrates was measured using steady-state fluorescence as previously described^17,57^. Binding affinities (*K*_*d*_) were obtained for double-stranded DNA substrates containing two different strand break termini (Supplemental Table S1). The fluorescence titration with the Gap1 substrate was carried out at room temperature. The titration with Gap2 was performed at 5 °C to avoid removal of the 3′-phosphate terminus.

### Circular dichroism spectroscopy

Far-UV circular dichroism (CD) measurements were performed with an Olis DSM 17 CD spectropolarimeter (Bogart, GA, USA), calibrated with a 0.06% solution of ammonium *d*-camphor-10-sulfonate. The temperature in the sample chamber was maintained at 20 °C. The CD spectra of wild-type and T323M-PNKP were measured as described previously^39^ and the results were analyzed according to the method of Chen et al.^38^.

### Fluorescence polarization

For direct binding by fluorescence polarization (FP), 20 nM of fluorescent-labeled XRCC4 peptide (GGYDES-pT-DEESKK) was mixed with different concentrations of PNKP, 6.8 nM to 13.9 μM for P101L-PNKP and, 7.4 nM to 15.2 μM for wild-type PNKP, in a reaction volume of 20 μL. The final buffer conditions were 22.5 mM Tris pH 8.0, 50 mM KCl, and 1 mM DTT. The fluorescence was recorded on a Perkin Elmer Envision plate reader (Waltham, MA, USA) at 538 nm with an excitation at 458 nm. The *K*_*d*_ values were determined using a plot of the polarization as a function of the log of protein concentration. Experiments were performed in duplicate or triplicate and plotted with error bars of one standard deviation.

### Cell culture

Human HeLa cells were obtained from Dr. David Murray (University of Alberta) and validated by ATCC cell line authentication service using short tandem repeat (STR) analysis. Cells were cultured in Dulbecco’s modified Eagle’s medium (DMEM)-F12 media supplemented with 5% fetal calf serum and 2 mM l-glutamine, incubated at 37 °C under 5% CO_2_ in a humidified incubator. All culture supplies were purchased from Invitrogen/Thermo Fisher Scientific (Waltham, MA).

### Quantitative real-time reverse transcription-polymerase chain reaction (qRT-PCR)

Whole RNA was extracted from the transient transfected cell lines (24 h after transfection) using the RNeasy Plus Mini Kit (Qiagen, USA) following the manufacturer’s protocol. Primers targeting the desired mRNA were designed through Primer-BLAST (NCBI). GAPDH forward: 5′-GTCTCCTCTGACTTCAACAGCG-3′, reverse: 5′-ACCACCCTGTTGCTGTAGCCAA-3′; PNKP forward: 5′-ATCCCAGCCAGATACTCCGC-3′, reverse: 5′-CTGCGGTGAACACTAGCAACT-3′. cDNA was reverse-transcribed with the High-Capacity cDNA Reverse Transcription kit (Applied Biosystems, USA). BrightGreen 2 × qPCR MasterMix-Low ROX (Abm, Richmond, BC) was used to perform the quantitative PCR process on QuantStudio 6 Flex Real-Time PCR Systems (Thermo Fisher Scientific, USA). The 2^−ΔΔCT^ method^60^ was applied by using GAPDH as the reference gene to calculate the PNKP mRNA level in all the transient transfected cell lines, compared with HeLa PNKP^−/−^ + WT-PNKP.

### Western blotting analysis

Western blotting was performed following a standard protocol^61^ with modifications. Antibodies used included PNKP antibody (sc-365724, Santa Cruz Biotech, USA), beta-actin antibody (sc-47778, Santa Cruz Biotech, USA), IR dye 800CW goat anti-mouse secondary antibody (926-32210, Li-COR Biosciences, USA). Results were visualized using an Odyssey Fc Imaging System (Li-Cor Biosciences, USA). Protein expression levels were calculated using beta-actin level as the reference protein.

### Establishment of transiently- and stably-transfected cells

The generation of PNKP-knockout HeLa cells by CRISPR technology has been previously described^25^. HeLa PNKP^−/−^ cells were plated in 60-mm dishes and transfected with plasmid constructs of interest using Turbofectin 8.0 following the manufacturer’s protocol. Transfected cells were incubated at 37 °C under 5% CO_2_ in a humidified incubator. Transiently-transfected cells were established after 24 h of transfection. Stably-transfected cells were established as follows: 24 h after transfection, cells were trypsinized and passaged at a 1:10 dilution in selective medium containing 800 µg/mL G418 (Thermo Fisher Scientific). Cells were expanded in selective media and GFP-positive cells were selected by fluorescence-activated cell sorting (FACS) (BD BioSciences, USA). Single cells with high to medium GFP intensity were picked and seeded into 96-well plates. After expanding selected single cells in G418 selective medium for one week, a high-content automated microscopy imaging system (MetaXpress Micro XLS, software version 6, Molecular Devices, Sunnyvale, CA) was used to select GFP positive single colonies. Positive individual colonies were later expanded in selective medium containing 800 µg/mL G418.

### Cellular localization of PNKP

For image acquisition, cells were plated on coverslips and fixed with 4% formaldehyde the next day. Immunofluorescence staining with tubulin antibody was performed to distinguish the cytoplasmic area. Nuclei were stained with DAPI. Cells were then placed on the stage of the Zeiss confocal LSM 710 microscope. Images were acquired using 40 ×/1.3 NA oil immersion objective.

### High-content screening

Widefield fluorescence images were taken with a high-content automated microscopy imaging system (MetaXpress Micro XLS, software version 6). Transient-transfected cells were plated in Greiner 96-well plates one day before imaging. Before acquiring images, cells were incubated with Hoechst 33258 (Sigma, cat. No. 94403) to a final concentration of 1 µg/mL for 20 min and then fed with fresh growth medium. At least 30 images (covering an area of ~ 2 mm^2^/image) per group were taken with a 10 × objective equipped with a siCMOS camera using bandpass filters (447–460 nm for Hoechst and 559–634 nm for GFP respectively). The images were analyzed with the MetaXpress Cell scoring module to compare each cell’s ratio between the area of the GFP protein distribution and the area of the nucleus (Hoechst staining). Each group yielded 4000–6000 cells with both Hoechst and GFP positive signals.

### Leptomycin B treatment

Stably-transfected HeLa PNKP^−/−^ cells were seeded on coverslips 24 h before treatment. In the leptomycin B treatment group, the growth medium of all cell lines was changed to medium containing 1 nM leptomycin B for 3 h. Control groups were changed with regular growth medium. After 3 h, the medium was removed and the cells washed with PBS 3 times. The cells were then fixed with 4% formaldehyde and stained with DAPI. Images were acquired using a 40 ×/1.3 NA oil immersion objective on a Zeiss confocal LSM 710 microscope.

### Crystal violet based viability assay

A crystal violet based assay was used for determining the viability of mutant cell lines after DNA damage as previously described^24^ with minor modifications. Briefly, 1 × 10^4^ cells were seeded in a 96-well plate and incubated for 24 h to enable adhesion. Cells were then exposed to 5, 10 or 15 Gy γ radiation (^60^Co Gammacell, AECL, Chalk River, ON, Canada), incubated for 24 h, washed with PBS, and stained with 0.5% crystal violet staining solution on a bench rocker. After 20 min, the plates were washed and air-dried. Methanol (200 µL) was added to each well and kept for 20 min at room temperature. The optical density of each well was measured at 570 nm (OD_570_) using a FLUOstar Omega microplate reader (BMG Labtech, USA), setting the average OD_570_ of the wells without cells as background, which was subtracted from each well. The average OD_570_ from the unirradiated cells was set as 100%. Cells were treated in quintuplicate in each group in each experiment; the experiment was performed three times independently.

### Alkaline single cell gel electrophoresis

Untransfected wild-type and PNKP^−/−^ HeLa cells, as well as stably transfected HeLa PNKP^−/−^ cells were exposed to 5 Gy γ radiation. 0, 1, 24 h after irradiation, 1 × 10^5^ cells were trypsinized and mixed with molten (37 °C) Comet LMAgarose (Trevigen, Gaithersburg, MD) at a volume ratio of 1:10. A 50-µL mixture was immediately pipetted onto comet slides (Trevigen). The slides were kept flat at 4 °C in the dark to allow the mixture to solidify, and then immersed in a 4 °C lysis solution (Trevigen) for 60 min. After that, the slides were immersed in a freshly made alkaline solution (300 mM NaOH, 1 mM EDTA) for 60 min at 4 °C. Slides were then placed in an electrophoresis apparatus filled with a freshly made alkaline solution before being subjected to electrophoresis at 1 V/cm and 300 mA for 40 min. The slides were then gently washed twice in distilled water for 5 min, immersed in 70% ethanol for 5 min and dried at 37 °C for 15 min. The slides were stained with 20 µg/mL ethidium bromide for 5 min then washed in distilled water. Images were acquired using an AxioSkop 2 Upright Fluorescence Microscope (Zeiss). For each time point, a minimum of 300 random cells from each group was analyzed using Comet Score 2.0 (TriTek Corp, Sumerduck, VA).

### Imaging of 53BP1 foci

Untransfected wild-type and PNKP^−/−^ HeLa cells, as well as stably transfected HeLa PNKP^−/−^ cells were exposed to 5 Gy γ radiation. 1, 6, 24 h after irradiation, cells were fixed and underwent immunofluorescence staining with 53BP1 antibody (Santa Cruz #sc-517281). Z-stack images were acquired using a Zeiss confocal LSM 710 microscope. For each time point, 120–200 cells from each group were analyzed using Imaris 9.5 software (Bitplane, Belfast, GB).

### Soft agar colony-forming assay

The protocol used was based on a published procedure^45^ with minor modifications. Briefly, the bottom layer added to wells in a 6-well plate consisted of melted 1% noble agar in pre-warmed 2 × medium (1:1 v/v), which was then allowed to set. 10^4^ untreated cells (un-transfected wild-type and PNKP^−/−^ HeLa cells, and stably transfected HeLa PNKP^−/−^ cells) were suspended in melted 0.6% agarose and pre-warmed 2 × medium mixture (1: v/v) to form the upper layer. After 14 days, 1 mL of 0.02% crystal violet was added to each well to stain the colonies. Four independent experiments were performed with at least 3 replicates each time.

## Supplementary Information

Supplementary Information 1.

Supplementary Information 2.

## Data Availability

All data are available in the main text or the supplementary materials.
