# Supplementary material for: Mutations of the DNA repair gene PNKP in a patient with microcephaly, seizures, and developmental delay (MCSZ) presenting with a high-grade brain tumor
Source: Sci Rep. 2022 Mar 30;12:5386. doi: 10.1038/s41598-022-09097-w (PMC8967877; doi:10.1038/s41598-022-09097-w)
Supplement: Supplementary file 1 — Supplementary Information 1. [file 41598_2022_9097_MOESM1_ESM.pdf]

Figure S2a: Protein levels of PNKP in transiently transfected cells

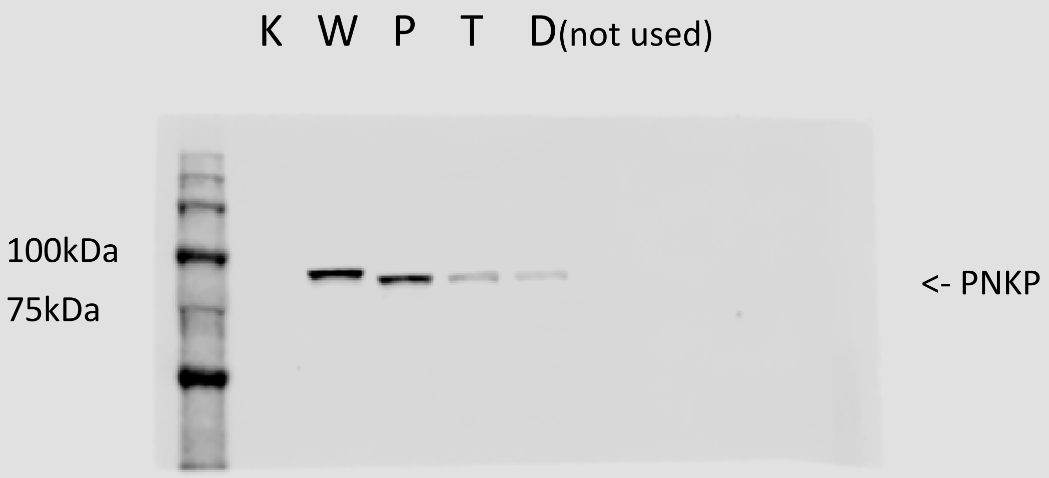

Supplemental Figure S2: Protein and mRNA levels of PNKP in transiently transfected cells (a) Western blot of transiently transfected cells, K - HeLa PNKP$^{-/-}$, W - HeLa PNKP$^{-/-}$ expressing wild-type PNKP, P - HeLa PNKP$^{-/-}$ expressing P101L PNKP, T - HeLa PNKP$^{-/-}$ expressing T323M PNKP. Gel has only one cut in between PNKP and Actin, they are cut from the same blot, which has a white space in between.

Figure S2a: Protein levels of PNKP in transiently transfected cells (*Showing Actin loading control*)

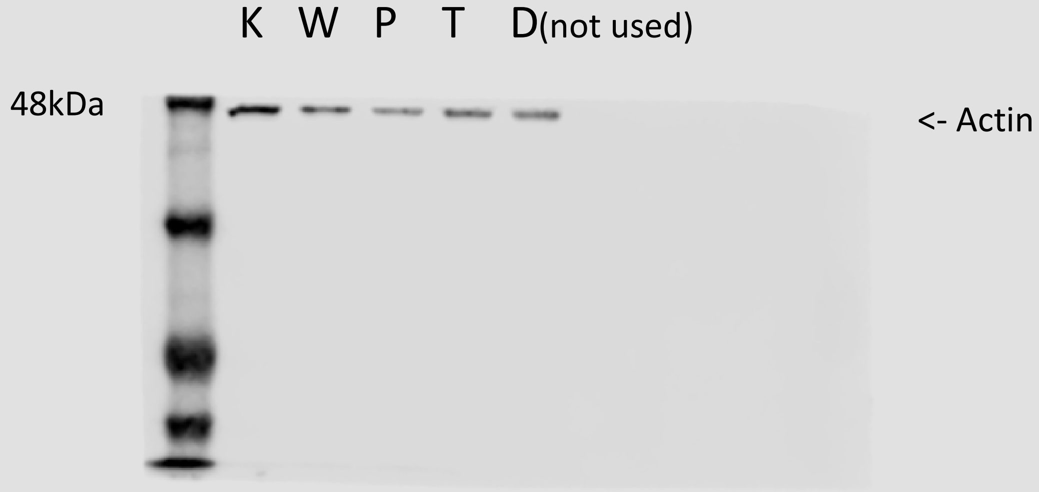

Supplemental Figure S2: Protein and mRNA levels of PNKP in transiently transfected cells (a) Western blot of transiently transfected cells, K - HeLa PNKP$^{-/-}$, W - HeLa PNKP$^{-/-}$ expressing wild-type PNKP, P - HeLa PNKP$^{-/-}$ expressing P101L PNKP, T - HeLa PNKP$^{-/-}$ expressing T323M PNKP. Gel has only one cut in between PNKP and Actin, they are cut from the same blot, which has a white space in between

Figure S2c *right*: mRNA levels of PNKP in transiently transfected cells

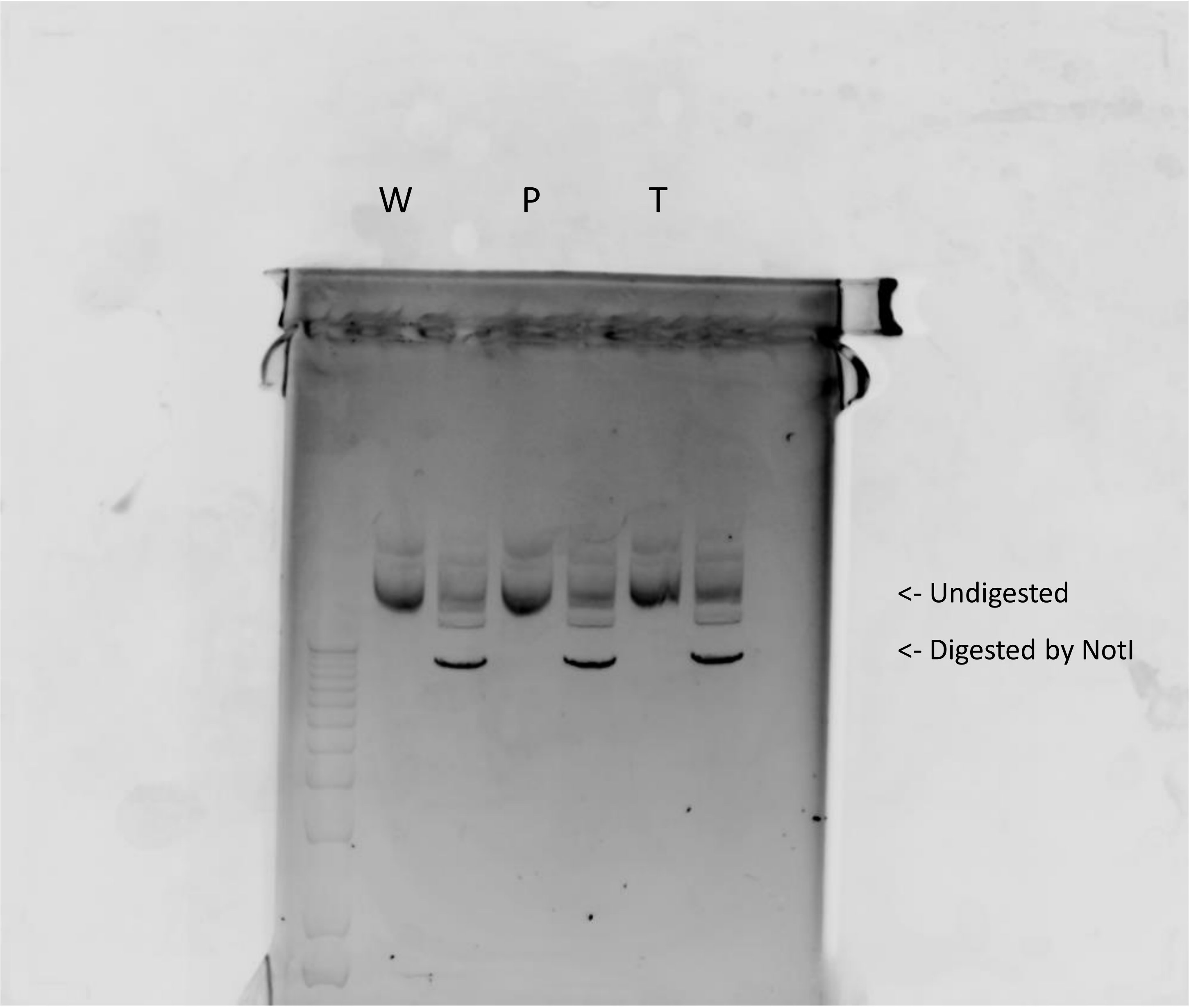

Supplemental Figure S2: Protein and mRNA levels of PNKP in transiently transfected cells. (c) DNA gel of the plasmid DNA (circular and linearized) used in the transfection showing equal quantities of the DNA constructs were used for the transfection. W - HeLa PNKP$^{-/-}$ expressing wild-type PNKP, P - HeLa PNKP$^{-/-}$ expressing P101L PNKP, T - HeLa PNKP$^{-/-}$ expressing T323M PNKP.

Figure S3: Western blot of stably transfected cells

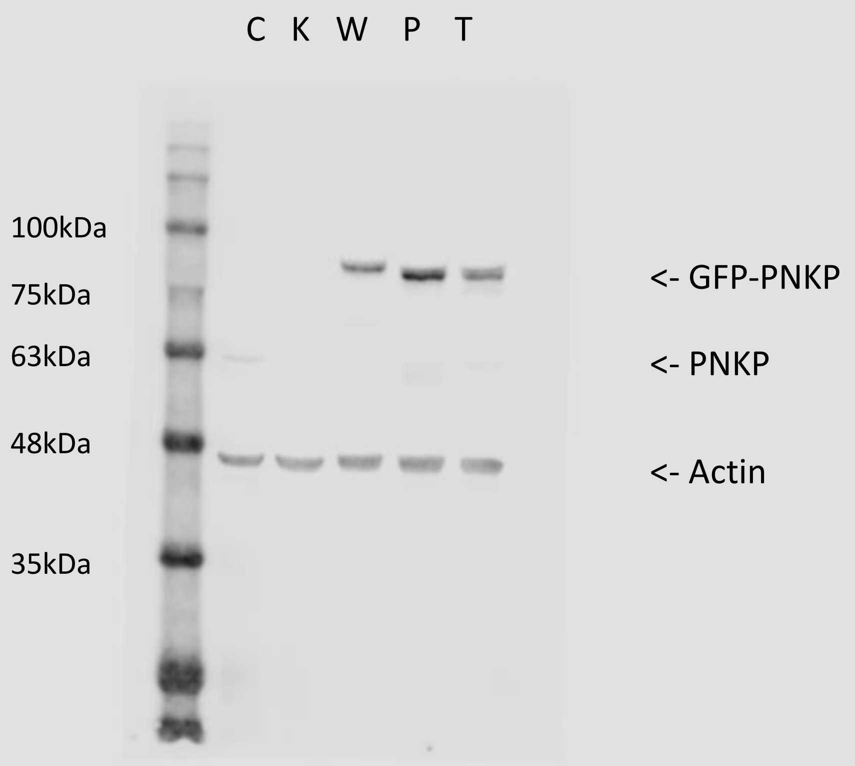

Supplemental Figure S3: Western blot of stably transfected cells. C – wild-type HeLa cells; K - HeLa PNKP$^{-/-}$ cells; W – HeLa PNKP$^{-/-}$ cells transfected with wild-type PNKP cDNA; P - HeLa PNKP$^{-/-}$ cells transfected with cDNA coding for P101L mutant PNKP; T - HeLa PNKP$^{-/-}$ cells transfected with cDNA coding for T323M mutant PNKP.

Figure S6b: Recombinant PNKP proteins

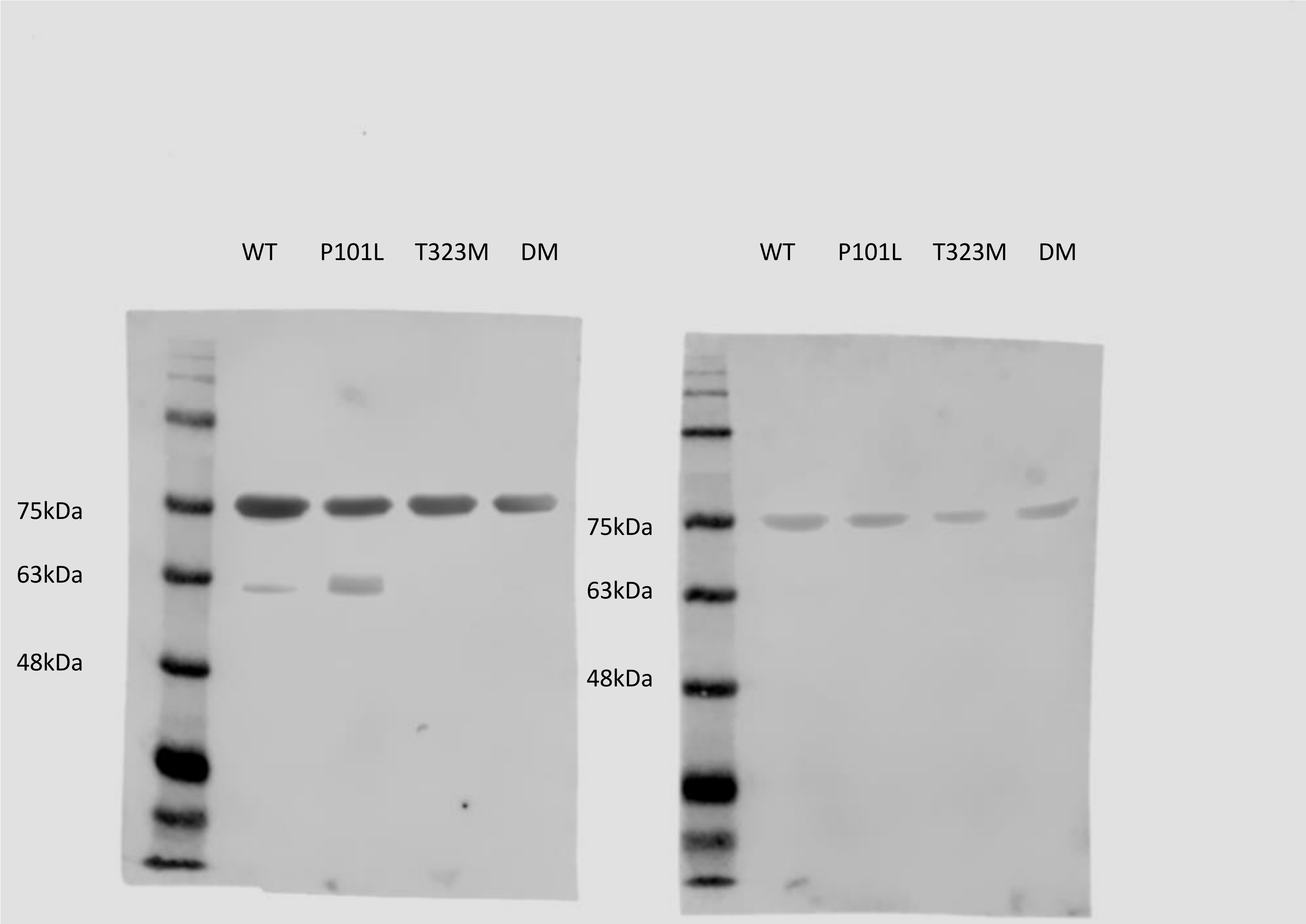

Supplemental Figure S6: Recombinant PNKP proteins. (b) Western blotting using PNKP antibodies, left: PNKP monoclonal antibody clone H101.2 (Fanta, et al. Hybridoma. 2001, 20:237-42. doi: 10.1089/027245701753179811); right: PNKP B-5 (Santa Cruz Biotech, sc365724).
