# Supplementary material for: Mutations of the DNA repair gene PNKP in a patient with microcephaly, seizures, and developmental delay (MCSZ) presenting with a high-grade brain tumor
Source: Sci Rep. 2022 Mar 30;12:5386. doi: 10.1038/s41598-022-09097-w (PMC8967877; doi:10.1038/s41598-022-09097-w)
Supplement: Supplementary file 2 — Supplementary Information 2. [file 41598_2022_9097_MOESM2_ESM.pdf]

Figure S1: Far-UV-CD spectra of wild-type (WT) and T323M PNKP

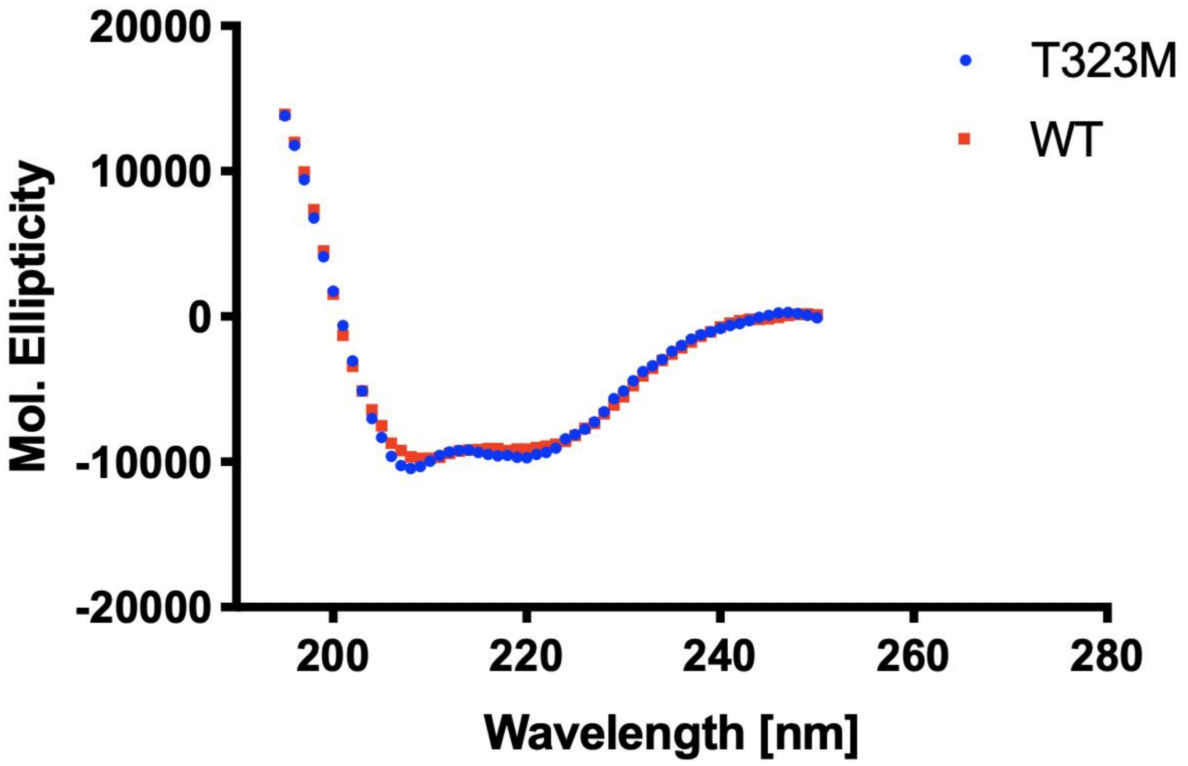

Supplemental Figure S1: Far-UV-CD spectra of wild-type (Wt) and T323M PNKP The concentration of PNKP was 0.5 mg/ml dissolved in 50 mM Tris, pH 7.5, 100 mM $NaCl_2$ and 1 mM DTT.

Figure S2: Protein and mRNA levels of PNKP in transiently transfected cells

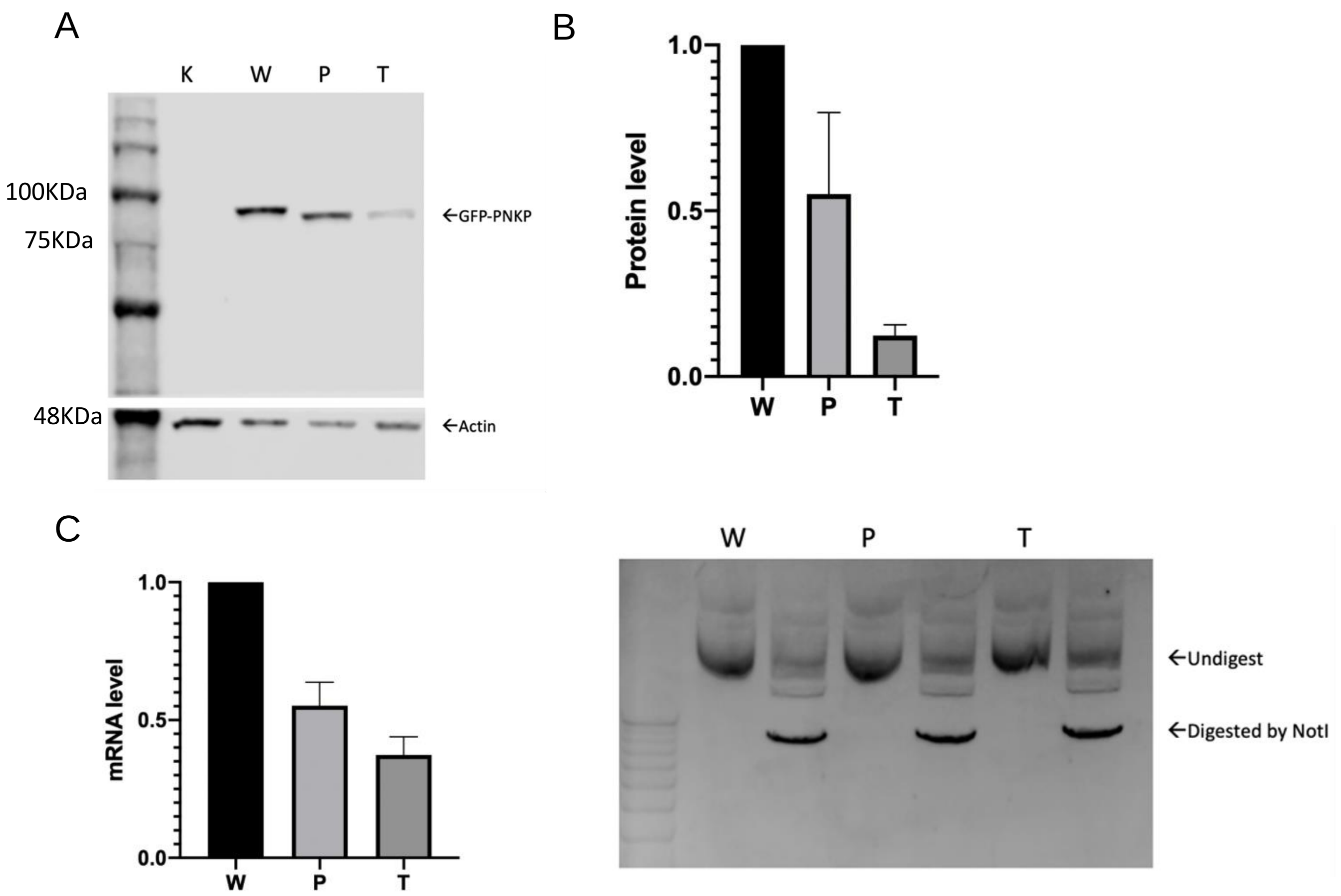

Supplemental Figure S2: Protein and mRNA levels of PNKP in transiently transfected cells (a) Western blot of transiently transfected cells, K - HeLa PNKP$^{-/-}$, W - HeLa PNKP$^{-/-}$ expressing wild-type PNKP, P - HeLa PNKP$^{-/-}$ expressing P101L PNKP, T - HeLa PNKP$^{-/-}$ expressing T323M PNKP. Gel has only one cut in between PNKP and Actin, they are cut from the same blot, which has a white space in between. (b) Relative levels of PNKP in transiently transfected cell lines were determined using beta-actin as the reference protein and normalizing the level of the PNKP in the HeLa PNKP$^{-/-}$ cells expressing wild-type PNKP to 1. Data represent the mean ± SEM of three independent experiments. (c) Left: Relative PNKP mRNA levels in transiently transfected cell lines. mRNA levels were calculated using GAPDH level as the reference with the level of the PNKP mRNA in the HeLa PNKP$^{-/-}$ cells expressing wild-type PNKP normalized to 1. Data represent the mean ± SEM of three independent experiments. Right: DNA gel of the plasmid DNA (circular and linearized) used in the transfection showing equal quantities of the DNA constructs were used for the transfection.

Figure S3: Western blot of stably transfected cells

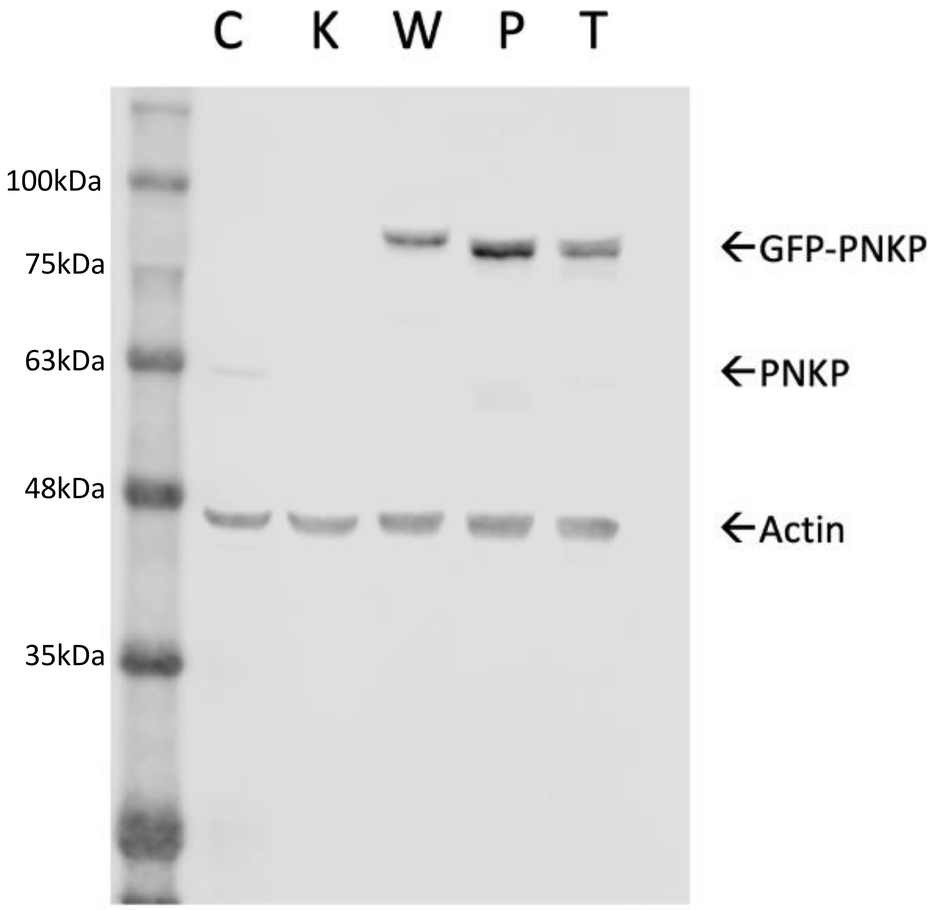

Supplemental Figure S3: Western blot of stably transfected cells. C – wild-type HeLa cells; K - HeLa PNKP$^{-/-}$ cells; W – HeLa PNKP$^{-/-}$ cells transfected with wild-type PNKP cDNA; P - HeLa PNKP$^{-/-}$ cells transfected with cDNA coding for P101L mutant PNKP; T - HeLa PNKP$^{-/-}$ cells transfected with cDNA coding for T323M mutant PNKP.

Figure S4: Cellular localization of PNKP

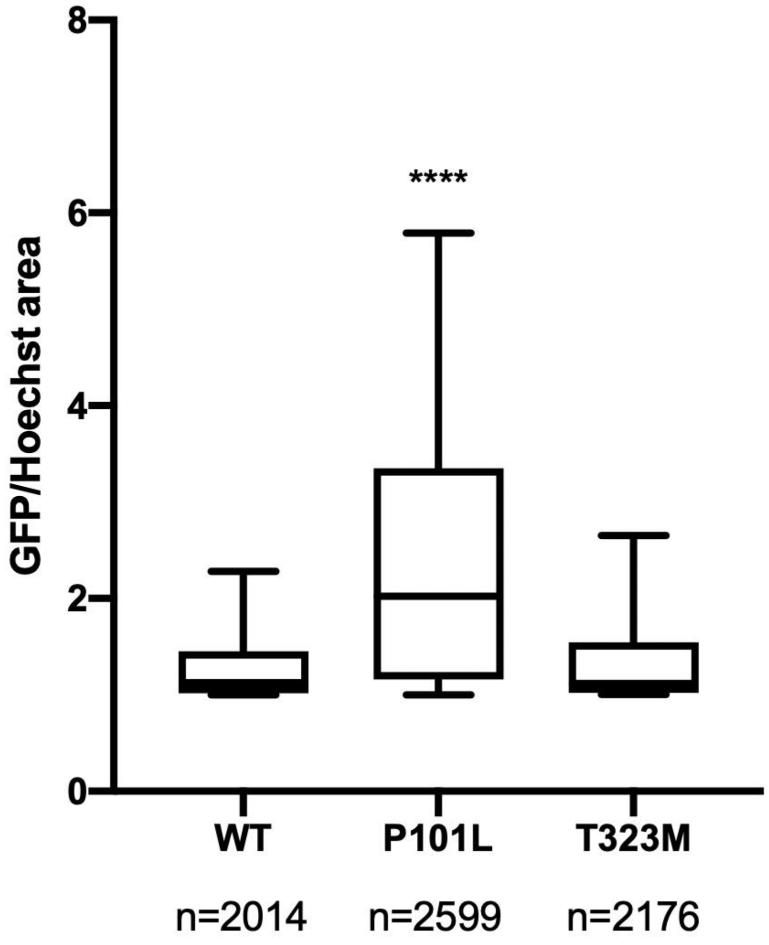

Supplemental Figure S4: Cellular localization of PNKP. (A) Nuclear vs cytoplasmic distribution of PNKP. The ratios were obtained by high-content analysis as described in Material and Methods. Whiskers indicate Min to Max ratio of GFP/Hoechst area in each group. 2% outliers were excluded by ROUT method, **** $P < 0.0001$.

Figure S5: Influence of *PNKP* mutation on cell transformation

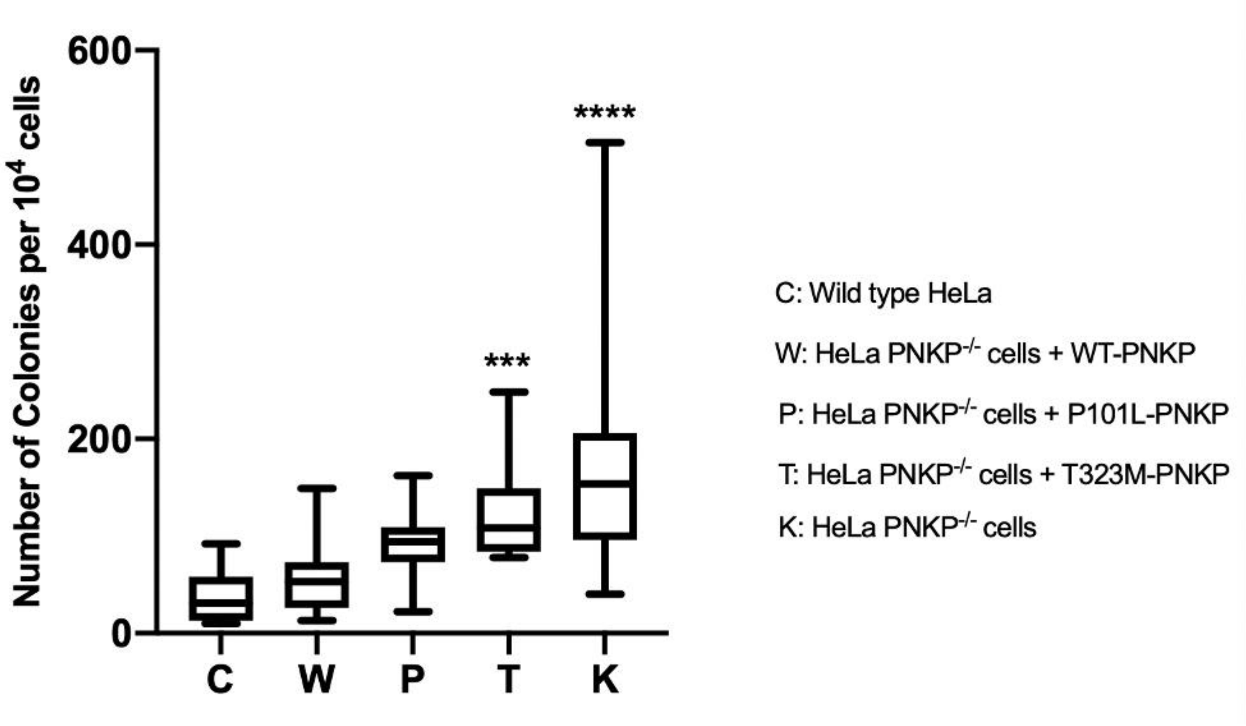

Supplemental Figure S5: Influence of *PNKP* mutation on cell transformation. The plot indicates the number of colonies generated by each cell type in soft agar two weeks after plating. Four independent experiments were performed with at least 3 replicates each time. Whiskers indicates Min to Max number of colonies per 10^4 cells. One-way ANOVA was performed to compare each outcome with the wild-type un-transfected HeLa cells using GraphPad Prism 7.0, GraphPad Software. P***<0.001, P****<0.0001.

Figure S6: Recombinant PNKP proteins

A

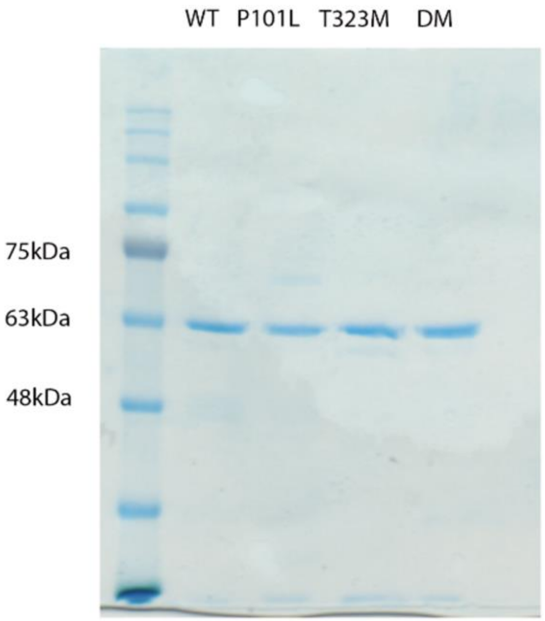

B

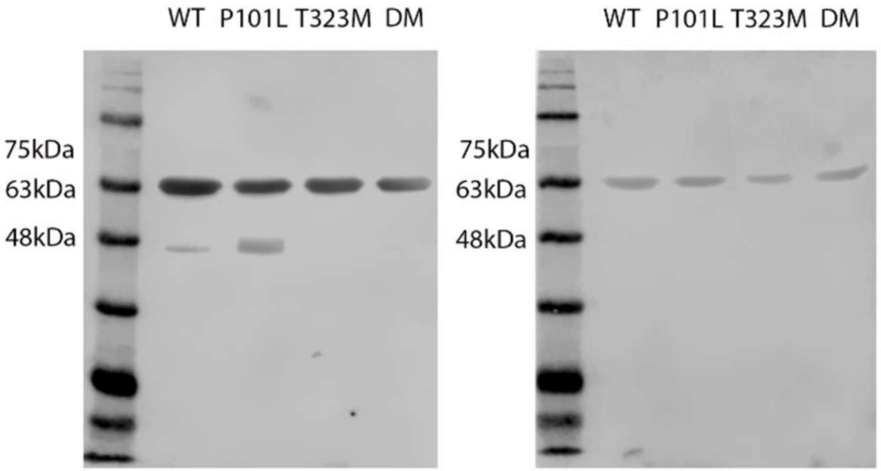

Supplemental Figure S6: Recombinant PNKP proteins. (a) Coomassie blue stain of purified PNKP proteins, from left to right lanes: wild-type (WT), P101L, T323M and double mutant (DM). (b) Western blotting using PNKP antibodies, left: PNKP monoclonal antibody clone H101.2 (Fanta, et al. Hybridoma. 2001, 20:237-42. doi: 10.1089/027245701753179811); right: PNKP B-5 (Santa Cruz Biotech, sc365724).

Supplemental Table S1: PNKP DNA binding activity

Binding affinity (K_d value, nM) between wild-type (WT) and mutant PNKPs and DNA substrates

	GAP1[a]	GAP2 (5ºC)[b]
WT	200±10	300±10
P101L	230±10	400±20
T323M	330±20	750±30
DM	300±20	550±30

Supplemental Table S1: PNKP DNA binding activity. The binding affinities (K_d) values were obtained by steady-state fluorescence. The determination with the GAP1 substrate was carried out at room temperature while determination with the GAP2 substrate was carried out in 5ºC.

Substrate sequence:

5’-pATTACGAATGCCCACACCGC GGCGCCCACCACCACTAGCTGGCC-3’

  3’-TAATGCTTACGGGTGTGGCGGCCGCGGGTGGTGGTGATCGACCGGp-5’

[a]GAP1 carried 3’- and 5’-OH termini at the gapped site.

[b]GAP2 carried 3’-phosphate and 5’-OH termini at the gapped site.

Supplemental Table S2: Secondary structural analysis of wild-type PNKP and T323M PNKP variant

Secondary structural analysis of Wild type PNKP and T323M PNKP

Sample	α-Helix (%)	β-Structure (%)	Random structure (%)
Wild type PNKP	39	31	30
T323M PNKP	37	33	30

Supplemental Table S2: Secondary structural analysis of wild-type PNKP and T323M PNKP variant

Supplemental Table S3: Identifying PNKP mutation induced novel nuclear export signals

A. Analysis of mutant PNKP by NetNES

Amino Acid and Location	ANN	HMM	NES
P101 (WT)	0.132	0.002	0.000
L101 (Mut)	0.433	0.008	0.195
T323 (WT)	0.073	0.002	0.000
M323 (Mut)	0.082	0.010	0.000

B. Analysis of mutant PNKP by LocNES

Position	Sequence	LocNES Score
88-102 (WT)	GVGDTLYLVNGLH***P***L	0.500
90-104 (WT)	GDTLYLVNGLH***P***LTL	0.650
87-101 (Mut)	LGVGDTLYLVNGLH***L***	0.663
88-102 (Mut)	GVGDTLYLVNGLH***L***L	0.628
90-104 (Mut)	GDTLYLVNGLH***L***LTL	0.768
315-329 (WT)	LNLGLPFA***T***PEEFFL	0.008
309-323 (Mut)	ADRLFALNLGLPFA***M***	0.015
315-329 (Mut)	LNLGLPFA***M***PEEFFL	0.011

Supplemental Table S3: Identifying PNKP mutation induced novel nuclear export signals (a). Individual scores of two mutation sites of PNKP by NetNES program [33]. ANN: Artificial Neural Network; HMM: Hidden Markov Models; NES: NES scores based on ANN and HMM value. (b). Possible NES candidates around the mutation sites, calculated by LocNES program [34]. P101L mutation increased the numbers of possible NES candidates from 2 to 3 with higher NES score; T323M mutation increased the numbers of possible NES candidates from 1 to 2 with similar low NES score.

Supplemental Table S4: Primers for site mutagenesis

Primers for site-directed mutagenesis

Primer name	Primer Sequence (5' to 3')
P101L Sense	5'-AATGGCCTCCACCTACTGACCCTGCGC-3'
P101L Antisense	5'-GCGCAGGGTCAGTAGGTGGAGGCCATT-3'
T323M Sense	5'-CCTGCCCTTCGCCATGCCTGAGGAG-3'
T323M Antisense	5'-CTCCTCAGGCATGGCGAAGGGCAGG-3'

Supplemental Table S4: Primers for site mutagenesis. Primers for site-directed mutagenesis to generate desired single point mutants. The double mutant was generated by using T323M primers on the P101L plasmid.
